# Dietary modulation of gut immunophysiology in food-producing animals: mechanistic links between the microbiota, mucosal immunity, and product quality

**DOI:** 10.3389/fphys.2026.1987339

**Published:** 2026-10-27

**Authors:** Ashfaq Ahmad Shah, Ayush Madan, Saranraj Pazhani, Deependra Pratap Singh, Iqra Nazir, Shikha Thakur, Renu Bhardwaj, Amit Gupta

**Affiliations:** 1 University Centre for Research & Development, Chandigarh University, Mohali, Punjab, India; 2 Department of Biotechnology, Parul Institute of Technology, Faculty of Engineering and Technology, Parul University, Vadodara, Gujarat, India; 3 Department of Microbiology, Sacred Heart College (Autonomous), Tirupattur, Tamil Nadu, India; 4 Department of Biosciences, Graphic Era (Deemed to be) University, Dehradun, India; 5 Department of Biotechnology, School of Applied and Life Sciences, Uttaranchal University, Dehradun, India; 6 Department of Zoology, University of Jammu, Tawi, Jammu & Kashmir, India

**Keywords:** animal product quality, dietary bioactives, gut immunophysiology, gut microbiota, mucosal immunity

## Abstract

In food-producing animals, the gastrointestinal tract is an immunometabolic interface where nutrients, microbial communities, epithelial barriers, and mucosal immune systems converge to regulate nutrient utilization, barrier integrity, host defense, and physiological adaptation. This review critically evaluates how dietary inputs are translated through the diet–microbiota–host axis into intestinal phenotypes, host–microbiota responses, animal performance, and animal-food characteristics. Evidence from ruminants as well as non-ruminants is synthesized, with emphasis on studies in food-producing animals that directly evaluate dietary interventions and mechanistic investigations linking dietary substrates with microbial metabolism, epithelial–immune interactions, and host immunometabolic responses. Evidence from non-livestock models is considered primarily to clarify biological mechanisms and is distinguished from livestock outcomes. Evidence indicates that dietary modulation can alter microbial ecology and metabolite production, thereby influencing epithelial sensing, mucosal immune regulation, redox homeostasis, inflammatory signaling, and cellular metabolism. Microbial metabolites, including short-chain fatty acids, indole derivatives, and bile-acid metabolites, emerge as intermediates linking nutritional inputs with epithelial and immune function. These processes provide mechanistic connections between intestinal homeostasis, nutrient partitioning, stress resilience, and, in some experimental contexts, productive phenotypes and animal-product characteristics. However, the magnitude and direction of these responses are context dependent and vary with species, gastrointestinal anatomy, developmental or production stage, dietary composition, microbial community structure, and environmental challenge. Mechanistic and intervention evidence is more developed in swine and poultry, whereas extrapolation to ruminants requires consideration of foregut fermentation and altered delivery of substrates and metabolites to the intestine. Importantly, alterations in microbial composition, barrier markers, or immune signaling should not be interpreted as evidence of improved production performance or product quality unless these outcomes are directly demonstrated. The review therefore distinguishes established livestock evidence from mechanistic inference, association, and proposed biological frameworks, while identifying unresolved causal relationships between gut processes and whole-animal phenotypes. Emerging approaches, including microbiome profiling, multi-omics, sensor-based phenotyping, and artificial intelligence, may support biomarker discovery and nutrition decision-making, but their predictive and intervention value requires prospective validation across species and production environments. Overall, current evidence supports the gut as a regulatory interface between nutrition and host physiology, while highlighting species-specific, contextual, and causal limitations that constrain predictive precision nutrition.

## 1 Introduction

The gastrointestinal tract (GIT) of food-producing animals is now known to be a key physiological nexus at which multiple dietary inputs, microbial metabolic activities, epithelial functions, mucosal immunity and host metabolic activities converge. In addition to its traditional digestive and absorption functions, the intestine is constantly absorbing luminal nutrients and microbial products while continuously integrating the luminal material with signals from the epithelial, immune, endocrine and metabolic compartments. The integration is crucial to ensuring the integrity of the barrier, immune tolerance, and efficient acquisition of nutrients and adaptation to environmental and infectious challenges. Thus, disruptions of intestinal homeostasis can impact nutrient utilization, inflammatory tone, disease response, and productive performance all at once, and gut immunophysiology is an important factor in animal health and production efficiency (Celi et al., 2017). As there has been a growing demand for efficient, sustainable and antimicrobial-responsible livestock production, there has been a considerable interest in nutritional strategies that can be used to alter gastrointestinal function. Therefore, alternatives or complementary modes to conventional nutritional and antimicrobial strategies have been investigated: probiotics, prebiotics, synbiotics, postbiotics, phytogenic compounds, polyphenols, organic acids, functional amino acids and fermentable substrates (Liu et al., 2024). But the biological effects of these interventions are not just determined by their chemical composition. Digestive system function, as well as microbial metabolism, epithelial sensing and host metabolism, can transform dietary substrates so that the physiological response to the dietary intervention relies on the interactions between the dietary intervention and the gastrointestinal ecosystem (Buonaiuto et al., 2025). This results in a very significant difference between the actual diet and the final biological signals fed to host tissues. Intestinal microbiota play an important role in this process. The substrates for microbial metabolism are determined by diet, while microbial activity produces metabolites that can alter epithelial, immune, endocrine and metabolic activity. Thus, short-chain fatty acids, indole derivatives, secondary bile acids, and other metabolites formed by intestinal microbiota may serve as intermediates in the cross-talk between the gut lumen and the host’s physiology (Koh et al., 2016; Yamada et al., 2016). The effects that are produced will depend on the context and could differ across species, gastrointestinal anatomy, developmental or production phase, basal diet, structure of the microbial community, environmental challenge and intervention characteristics. Therefore, changes in microbial levels and/or diversity must not be used alone as an indicator of better intestinal function or productive performance. At the host interface, the sensing of microbial and dietary signals is mediated by epithelial and immune sensing systems that modulate barrier function, inflammatory responses, immune tolerance and tissue repair. Response to luminal microorganisms and their products is governed by pattern-recognition and metabolic sensing pathways, as well as epithelial–immune communication (Peterson and Artis, 2014). Thus, the intestinal barrier is not a single, static anatomical barrier but a complex, dynamically functioning system, which includes the integrity of the epithelial barrier, mucus production, organization of the junctions, antimicrobial function, renewal of the cells, and immune surveillance (Thaiss et al., 2016). The modulation of these processes by nutrition may affect the physiological adaptations and inflammatory dysfunction balance. Likewise, nutrient availability and inflammatory signaling are linked by immunometabolic pathways that control cellular energy utilization, redox homeostasis, proliferation, repair and immune-cell function (Turner, 2009). The interrelated mechanisms offer a biological underpinning for the relationship between dietary interventions and intestinal resilience, with evidence for downstream production effects being inconsistent in certain animal species and experimental settings. Another question that remains unanswered is how well the positive changes in gut phenotypes relate to measurable whole-animal and product qualities. Changes in the function of the intestine can affect nutrient partitioning and systemic metabolic responses, which in turn can influence growth, feed efficiency, reproduction, lactation, muscle deposition and meat, milk and eggs composition or quality. However, correlations between gut microbiota profiles, immunity markers, barrier measurements, and productivity parameters cannot be interpreted as causes. To show a true gut-to-product pathway, the intestinal physiology and the activity of the microbes, the production of metabolites, the immune phenotyping, partitioning of nutrients, and direct measurements of product characteristics should be integrated. Such integration could be enabled by the use of multi-omics and advanced phenotyping techniques, which still have some potential for predictive or intervention-level functionalities but are emerging fields that need to be validated across species and production environments (Jami and Mizrahi, 2012).

### 1.1 Knowledge gap and rationale

There are many reviews on intestinal health, gut microbiota, mucosal immunity, feed additives or individual nutrition interventions in livestock, but these literatures are perceived as somewhat distinct fields. The critical connection between dietary substrates and microbial metabolism, epithelial and immune responses, systemic physiological adaptations, and ultimately animal-product phenotypes is relatively underdeveloped. Furthermore, the degree of evidence for the mechanisms varies between food-producing species and biological mechanisms found in rodents, humans, or cellular systems do not necessarily apply in the same way or to the same extent in pigs, poultry, ruminants or other food-producing animals. A big difficulty is thus the differentiation of experimentally proven livestock mechanisms, mechanistic evidence from other experimental systems, and biological hypotheses that still need to be tested in production situations. Against this backdrop, this review critically discusses dietary modulation of gut immunophysiology in food-producing animals in a hierarchical sequence, from input to microbial ecology and metabolites to epithelial and mucosal immune responses, to systemic physiology and resilience, to the phenotype of animal products. Mechanistic links between microbial metabolism, intestinal barrier function, mucosal immune regulation, immunometabolism and oxidative–inflammatory homeostasis are highlighted, with special focus on evidence for impact on production efficiency and the quality of meat, milk and eggs, across experimental strength and species context. After laying the groundwork for understanding the physiological and immunological aspects of the gastrointestinal system, the review explores the diet–microbiota–immune axis, regulation of the intestinal barrier, oxidative and inflammatory processes, and the gut–resilience–product quality axis. Next, emerging applications of microbiome-informed nutrition and precision approaches are considered as prospective solutions. The integrated framework of dietary intervention and gut microbial ecology, epithelial barrier function, mucosal immunity, immunometabolic responses, animal resilience and product quality is summarized in **Figure 1**.

**Figure 1:**
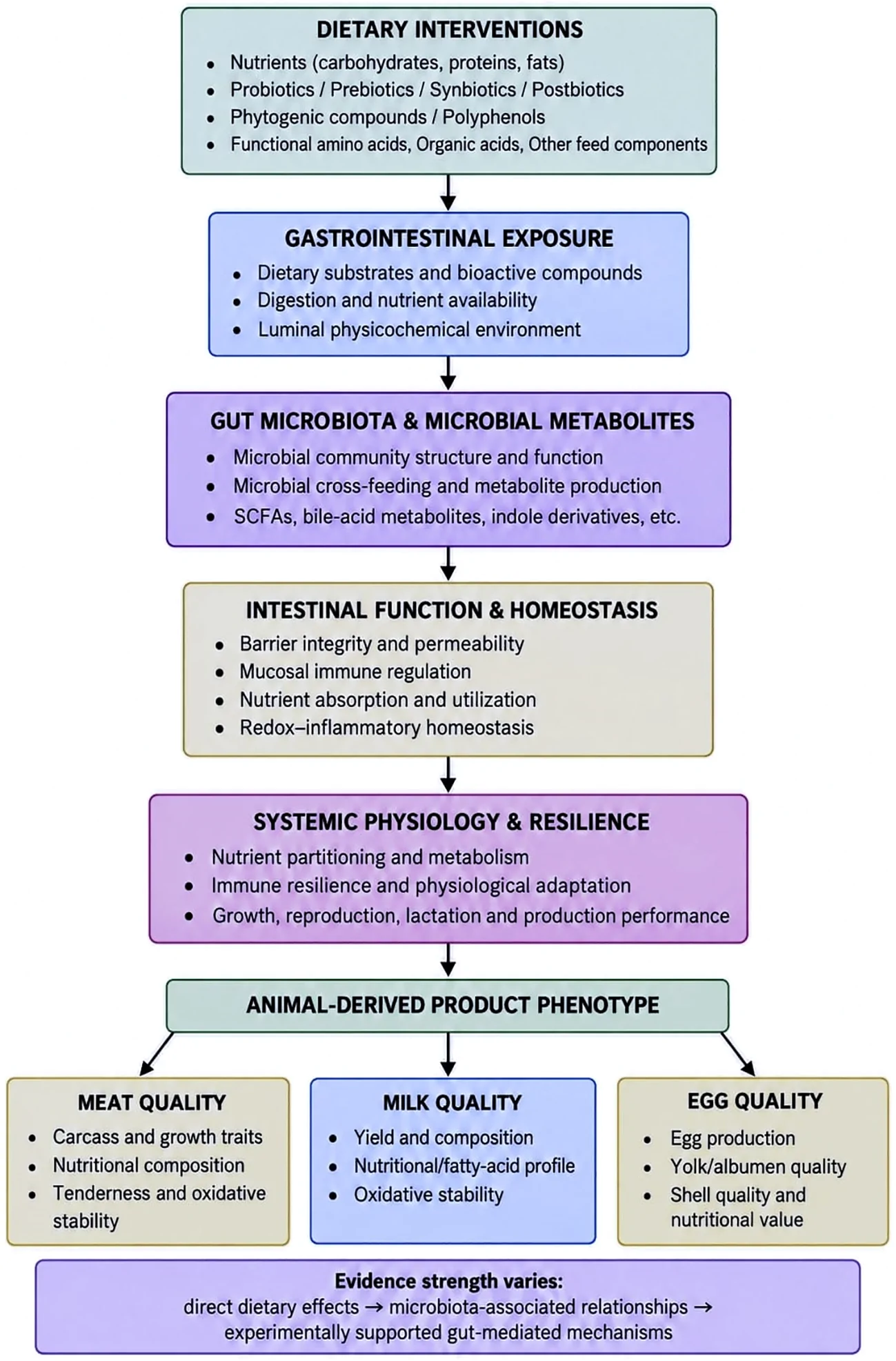
Dietary modulation of gut immunophysiology: from dietary signals to animal product quality.

### 1.2 Literature search and evidence synthesis

A systematic literature search was conducted to find evidence on the link between dietary inputs, gastrointestinal function, microbial ecology and immunophysiological responses in food-producing animals. Literature was obtained using the PubMed/MEDLINE, Scopus, Web of Science and Google Scholar databases (data published from January 2009 to August 2026). Search terms included “dietary intervention”, “feed additive”, “gut”, “gastrointestinal”, “microbiota”, “microbiome” and “microbial metabolites” and limited to food-producing animals. Literature references on dietary modulation of gastrointestinal microbial and physiological processes were identified by using combinations of these. The evidence base comprised dietary intervention studies, feed-additive studies, animal experiments, observational investigations, mechanistic studies, microbiome and multi-omics investigations and relevant experimental-model studies found through the searches. When assessing effects on intestinal function, mucosal immunity, physiological resilience, production performance, or quality of animal products, priority was placed on studies that were done directly in food-producing animals. Published literature prior to 2009 was selectively included to provide background on key concepts or mechanisms in biology. Evidence was examined in reference to the experimental context. When making decisions about livestock physiology, dietary response, production characteristics, or product quality, direct evidence from food-producing animals was given priority. Livestock observational studies were used to interpret findings as being associations rather than causalities when experiments did not provide evidence of casualty. Mechanistic context and biological plausibility were provided mainly by evidence from rodents, humans, cell culture or other experimental systems, which were not considered as evidence for food-producing animals. If a mechanism had not been well demonstrated in livestock, it was stated to be a mechanism that needs to be validated or a hypothesis that needs to be validated before it can be used as a livestock production-animal response. When drawing conclusions about the results of the studies across pig, poultry, ruminant, and other food-producing species, special attention was paid to species, gastrointestinal anatomy, physiological or production stage, dietary background, environmental conditions, and experimental challenge. Changes in microbiota composition, microbial metabolites, barrier-related markers, or immune parameters, were not necessarily automatically concluded as production and/or product improvements unless assessed directly. This allowed the review to identify mechanisms that were well established, livestock associations, mechanisms that were validated from other experimental systems, and hypotheses that needed to be tested. This synthesis was structured around the central theme of inputs in the diet, microbial ecology and metabolites, epithelial and mucosal immune response, systemic physiology and resilience, and animal-product phenotype. This approach was used to bring together evidence for gastrointestinal physiology, microbiology, mucosal immunology, immunometabolism and animal nutrition, and minimize unwarranted inter-species and inter-experimental extrapolation.

## 2 Fundamentals of gut immunophysiology in food-producing animals

The GIT of food-producing animals functions as a coordinated physiological unit where digestion, sensory responses to nutrients, protective epithelial functions, microbial containment, immune monitoring, and tissue repair are interdependent processes. Its fundamental physiological challenge is to sustain adequate nutrient supply and absorption, while at the same time continuously dealing with the presence of nutrients and exposure to nutritional antigens, commensals, the environment, and pathogens. Gut homeostasis thus reflects a dynamic interplay between the mutually opposed requirements for immune tolerance and host resistance, epithelial barrier function and restricted permeability, microbial coexistence and limitation, and inflammatory activation and resolution. Instead of discrete functional units, epithelial, neural, immune, and metabolic axes interact in a network to coordinate gastrointestinal activity with nutrient supply and environmental pressures (Kogut and Arsenault, 2016). This organization is strongly influenced by species-specific gastrointestinal anatomy and fermentation physiology; consequently, microbial and immunophysiological responses observed in hindgut-fermenting monogastrics cannot be assumed to be equivalent to those occurring in foregut-fermenting ruminants or in poultry. Accordingly, subsequent mechanistic interpretations distinguish direct evidence from food-producing animals from associations and evidence derived from other experimental systems. This integrated organization serves as a physiological platform upon which dietary manipulation of the overall animal’s welfare, resilience, and productive efficiency can be exerted.

### 2.1 Intestinal architecture as an immunophysiological interface

The intestinal epithelium is the site of direct interaction between the luminal environment and the body tissue. The main constituent epithelial cell population is represented by enterocytes. These absorptive cells manage absorption in close coordination with nutritive, metabolic, and protective roles. Goblet cells are engaged in mucus synthesis and secretion and various other functions. Enteroendocrine cells signal a variety of absorbed, microbially derived inputs to nearby cell types via endocrine and paracrine mechanisms. Intestinal stem cells are responsible for continuous epithelial renewal. There are other epithelial cell types that provide defense, contribute to immune communication, and promote tissue repair. These cell types demonstrate their importance through their integrated response to lumen- or tissue-derived messages rather than acting totally in isolation (Heppert et al., 2021). The epithelial surface is encased in a high- mucus extracellular matrix, which provides a physical barrier to further microorganisms and reduces the extent of contact between them and the epithelial cells. Underneath the mucus barrier, junctions at the apical lateral border of epithelial cells help control the transfer of small molecules, solutes, and macromolecules through the intercellular space, creating a selective paracellular pathway yet preventing an unrestricted leak of luminally contained substances. The ability of epithelium itself is vast, having sensors and effectors; epithelial cells can secrete antimicrobial molecules, cytokines, chemokines and mediators and regulate themselves according to exogenous stimuli coming from microbes or the underlying tissue and are then active sensors and regulators in maintaining homeostatic conditions. Epithelial structure is intrinsically dynamic. Availability of nutrients, metabolites produced by microorganisms, inflammatory mediators, signals of oxidative stress, thermal insult, and an infectious challenge to epithelia affect proliferation, differentiation, transport processes, and restoration. Disruptions in this homeostasis can predispose to heightened exposure to intraluminal antigen/products of microbial products to the underlying immune cells, or it is equally possible for erroneous inflammatory signaling per se to affect normal function, metabolism, and structural organization (Kurashima and Kiyono, 2017). Detailed mechanisms governing tight-junction regulation, mucus protection, permeability, and nutritional preservation of the barrier are considered separately in Section 4.

### 2.2 Gut-associated lymphoid tissue and mucosal immune surveillance

In food-producing animals, the intestinal immune system is one of the biggest and continuously challenged immune compartments. Gut-associated lymphoid tissue (GALT) is constantly monitoring the contents of the lumen and tolerating the dietary and intestinal flora. GALT, along with cells scattered throughout the lamina propria and epithelium, continually monitors the contents of the lumen and tolerates the presence of food and microorganisms in the gut. This involves subtle discrimination between the signals of the physiology of microorganisms and those of infection and tissue damage, as well as the signals of nutrition. Epithelial cells, macrophages, dendritic cells, and other cells of the immune system express pattern-recognition receptors (PRRs) such as Toll-like receptors (TLRs) and NOD-like receptors (NLRs), as well as other cells that sense microbial products, which represent the first line of host defense (Shah et al., 2025). When PRRs recognize microbial-associated molecular patterns (MAMPs), they trigger pathways that can modulate antimicrobial responses, cytokine production, antigen presentation and tissue repair. Importantly, these responses are context-dependent in nature, as opposed to being uniformly pro-inflammatory, and must therefore have mechanisms to prevent inappropriate inflammation while also maintaining rapid responses to pathogens upon continuous exposure to commensal-derived signals. Macrophages clear microbes, monitor them, undergo efferocytosis and regulate inflammation in the intestine, and dendritic cells internalise luminal or epithelial-associated material and are involved in antigen presentation and adaptive immune programming (Shah et al., 2021). Adaptive immunity then regulates for longer periods of time against the same antigen using secretory immunoglobulin A (sIgA), populations of T-cells, and other subsets of lymphocytes. ILCs represent another fast conduit between epithelial signals and barrier defence/repair, mediated by cytokines. Secretory IgA has the capacity to inhibit the adherence and invasion of microorganisms, and to facilitate immune exclusion with relatively low inflammatory damage. CD4 T cell subsets, such as Th1, Th2, Th17, and Treg cells, play different roles in pathogen control, epithelial support, and tolerance, the balance of which is responsible for mucosal homeostasis (Mantis et al., 2011). These immune compartments are not organized and do not have the same functions in all food-producing species. The intensity and site of immune–microbial interactions could vary due to differences in gastrointestinal anatomy, microbial compartmentalization, mucosal architecture, developmental stage, and production status. As such, conserved mechanisms, including PRR signalling, sIgA production, ILC responses, and Th17/Treg regulation, offer a shared immunologic axis, but their physiological implications need to be understood in the context of the individual livestock species. In particular, results from general mammalian or experimental systems should not be assumed as demonstrated outcomes in pigs, poultry or ruminants. Over- or under-activation of the immune system can lead to tissue damage and/or increased metabolic consumption, while the opposite can weaken the ability to resist pathogens. This means that intestinal immune competence relies not on the highest possible level of immune activation, but rather on an appropriately damped level of detection, response, and resolution. Dietary interventions can therefore affect animal health by modulating this immune calibration, but alterations in immune markers cannot be equated to improvement in gut function and/or production performance.

### 2.3 Epithelial–immune communication and mucosal homeostasis

Intestinal epithelial cells are in close contact with the immune system and have an extensive communication network with it. When epithelial cells encounter microbial products or stresses, they synthesize and release cytokines, chemokines, antimicrobial molecules and other mediators that control the recruitment of leukocytes and the development and function of recruited leukocytes. Meanwhile, the cytokines that are secreted by immune cells control epithelial proliferation, differentiation, antimicrobial activity and repair. This reciprocal signaling incorporates the lumen information with information from host tissues in the mucosa (Soderholm and Pedicord, 2019). Importantly, the epithelial–immune communication is bidirectional, not a single, unidirectional epithelial or immune response, and the outcome depends on the nature, intensity and duration of the stimulus in the lumen or in the tissue. Thus, the communication between epithelial cells and immune cells mediated by cytokines like IL-25, IL-33, TSLP, IL-22, interferons, or pro-inflammatory cytokines (TNF, IL-1 family members) are highly context-dependent. For example, in certain contexts, IL-22 can promote epithelial repair and antimicrobial immunity, while chronic or unchecked inflammatory signaling can lead to barrier dysfunction and tissue pathologies. Likewise, activation of immune cells can also affect the activity of epithelium-associated nutrient transport and metabolism, suggesting that mucosal immunity is not independent of gastrointestinal physiology. The idea of immune tolerance has a mechanism (reciprocal signaling): Responses to dietary antigens and commensals are regulated, but the mucosa is still able to respond quickly when the biology of the microbes or tissue microenvironment suggests a biological threat. Such relationships, however, should not be equated with being equally beneficial and the same in all food-producing species, since the size of these relationships and their physiological effects could differ depending on gastrointestinal anatomy, microbial exposure, developmental status, and inflammatory challenge. The following “cautious immune surveillance” has become an “imprint” of gut immunophysiology (Didriksen et al., 2024).

### 2.4 Neuroendocrine and immunometabolic integration

Regulatory responses mediated by the nervous and endocrine systems are interwoven with gastrointestinal immune responses. Within the gastrointestinal (GI) epithelium, enteroendocrine cells monitor the availability of nutrients and luminal factors, and produce hormones and other signaling molecules, which in turn modulate GI motility and fluid secretion, feeding behavior, and systemic metabolism. The enteric nervous system receives signals from epithelial, microbial, immune and metabolic, and neuronal pathways allow for two-way communication between the intestine and the rest of the organism. These pathways are thus an extra mechanism by which nutrition and environmental factors can impact gastrointestinal function (Sharkey and Mawe, 2023). Importantly, the neuroendocrine integration that occurs here is not an independent regulatory pathway, but rather an integration between luminal sensing and whole animal physiological adaptation. Metabolism at the cellular level has a profound effect on the functional state of immune cells. The mechanisms underlying the effects of nutrients, cellular energy state, oxygen tension, and inflammatory cytokines converge on pathways involving mechanistic target of rapamycin (mTOR), AMP-activated protein kinase (AMPK), phosphoinositide 3-kinase/protein kinase B (PI3K/Akt), and hypoxia-inducible factor-1α (HIF-1α). These pathways control cell proliferation, mitochondrial activity, energy distribution, antimicrobial activity, cytokine production and tissue repair. Therefore, whenever the immune system is activated, there is an metabolic cost to the organism in the form of substrate redistribution and energy expenditure for defence and restoration. This association is especially important in food-producing animals as resources used for sustained immune activation could be used for other production functions such as growth, reproduction, lactation, egg production, muscle deposition, and other production functions (Loor and Elolimy, 2022). The link between immune activation and production efficiency is not always a linear one, however; a properly functioning immune system can also reduce the metabolic load of chronic inflammation while an underactive immune system can also lead to greater susceptibility of disease and impact performance. In this way, a well-regulated immune response can be beneficial for production efficiency, as it can avoid unnecessary expenditure of inflammatory resources and maintain an effective immune response to the host. The interactions between diet derived signals and microbial metabolites with immunometabolic processes are discussed in detail in Section 3.

### 2.5 Gut homeostasis, resilience, and physiological adaptation

Ultimately, gut homeostasis must be viewed as an equilibrium state rather than a static physiological condition. As food is variable in composition and quantity, nutrient supply is unpredictable, the presence of microorganisms and pathogen attack is persistent, and temperature, management practices, and numerous other factors vary, the healthy gut must continually respond. To that end, resilience will depend upon the intestinal tract to restore or maintain epithelial integrity, microbial status, immune function, metabolic capabilities and overall tissue function upon provocation. Many factors, including rapid turnover of the epithelia, controlled immune tolerance, efficient antimicrobial defenses, orderly inflammatory resolution, metabolic plasticity and coordinated cross-compartmental communication of the intestine, all play a role in this robustness. Nonetheless, it is important to note that resilience is not the same as being a fortress against all intrusions (Kaore et al., 2025). Although a durable gastrointestinal system can undergo acute physiological perturbation, it must retain the ability to restore homeostasis without advancing to chronic barrier dysfunctions, microbial community disruption or continuous inflammation. In food-producing animals, such ability is related directly to productivity and health. Stimuli such as environmental stresses or infective agents can modulate feed intake, intestinal permeability, the activity of the immune system, and the allocation of nutrients; this will eventually lead to elevated maintenance costs and decreased production efficiency. In contrast, maintaining GIT integrity, in particular, improves nutrient partitioning and physiological status under challenging circumstances. This makes the gut a key factor in the animal’s overall resilience (Shin et al., 2019). The principal structural, cellular, immunological, neural, and metabolic components that collectively constitute gut immunophysiology in food-producing animals are summarised in **Figure 2**.

**Figure 2:**
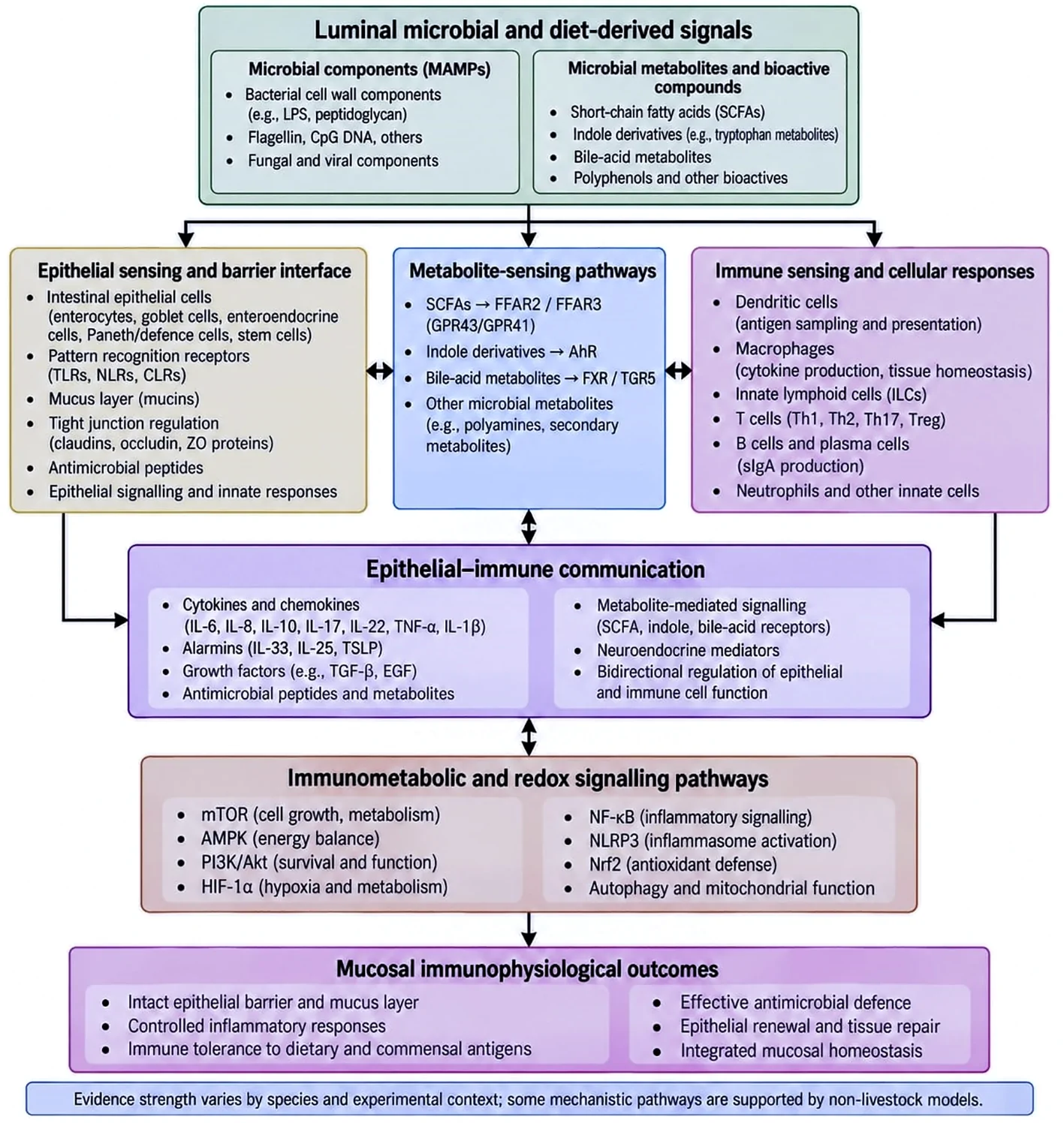
Fundamental organization of gut immunophysiology in food-producing animals, illustrating the coordinated interaction among intestinal epithelial architecture, mucosal immune surveillance, epithelial–immune communication, neuroendocrine regulation, immunometabolic signaling, and physiological resilience.

### 2.6 Species and physiological context

While general principles of gut immunophysiology remain conserved among most food animals, their respective contributions to total immunity or the pattern in which they manifest are relative and function depending on gastrointestinal structure, feeding strategies, phase of development, reason for production, the composition of microbiota, and exposure. Monogastric organisms like pigs and poultry have a gastrointestinal organization, whereby rapid intestinal passage, fermentation, and enteric surveillance are strongly interacting system components. In addition, ruminants have a pre-gastric fermentative system with major impact on the conversion of nutrients, availability of microbial metabolites, and intestinal conditions at later stages in the tract. As a consequence, intervention that results in a given response in an individual’s microbes, metabolism, or immune status does not necessarily predict the same response in another species. There is also relevance of physiology- 4 the responses to a dietary manipulation might well differ based on whether an individual is old or young, pregnant or non-pregnant, high- or low-producing, starving or well-fed, and indeed on its body temperature, pathogen history, and existing microbiomic structure (Li et al., 2025). Therefore, the biological efficiency of dietary treatment must be considered and adjusted for the physiological state where it will be utilized, rather than the inherent ability of the dietary substance itself. In the evidence synthesized in this review, direct dietary–immune and barrier findings are most extensively represented by studies in pigs and poultry. For example, pig studies have demonstrated effects of probiotics, dietary glutamate, α-ketoglutarate, dietary fibre, and isobutyric acid on intestinal immune, barrier, microbial, or metabolic outcomes, whereas poultry studies provide evidence for probiotic, phytogenic, postbiotic, and antioxidant-related modulation of intestinal function and inflammatory or redox pathways. By contrast, the ruminant evidence considered here is used primarily to establish species-specific gastrointestinal and fermentation context rather than to infer equivalent responses to the interventions demonstrated in pigs or poultry. Accordingly, mechanisms identified in monogastric animals are not assumed to produce equivalent outcomes in ruminants (Didriksen et al., 2024; Li et al., 2025). Taken together, these principles define gut immunophysiology as a homeostatic system in which epithelial, microbial, immune, neural and metabolic events cooperate to control intestinal function and whole animal adaptation. Since diet constitutes one of the most significant components of the intestinal luminal milieu, the subsequent question is how diet-derived substances impact the microbial ecosystem and its metabolic function, and in turn how such changes translate to physiological and immune responses from the host. The “diet-microbiota-function” cascade described herein is the mechanistic core in the section hereafter. A side characterisation in terms of major constituents, components of the system on the molecular level, physiology and nutritional dependence of these entities is available in **Table 1**.

**Table 1:** Major components of gut immunophysiology and their functional roles in food-producing animals.

Gut component/system	Principal cellular or molecular elements	Major physiological function	Immunological function	Nutritional/dietary sensitivity	Relevance to animal health and productivity	Representative references
Intestinal epithelium	Enterocytes, goblet cells, Enteroendocrine cells, intestinal stem cells	Nutrient absorption, epithelial renewal, sensing of luminal nutrients and microbial products	Provides first-line innate defense; produces cytokines, chemokines and antimicrobial molecules	Nutrient availability, amino acids, fatty acids, phytochemicals, microbial metabolites	Determines absorptive capacity, barrier resilience and tissue repair	(Allaire et al., 2018; Du et al., 2023)
Mucus layer	MUC2 and other mucins, glycoproteins, antimicrobial peptides, secretory IgA	Physical separation of luminal microorganisms from epithelial surfaces; lubrication	Immune exclusion and regulation of host–microbe contact	Dietary fibre, fermentable carbohydrates, microbiota-derived metabolites and phytochemicals can influence mucus production/function	Protects epithelium and reduces microbial translocation and inflammatory burden	(Gustafsson and Johansson, 2022)
Tight-junction barrier	Claudins, occludin, ZO-1 and other zonula occludens proteins, junctional adhesion molecules	Regulation of paracellular permeability and epithelial integrity	Restricts passage of microbial products, antigens and toxins into mucosal tissues	Sensitive to dietary amino acids, fatty acids, polyphenols, microbial metabolites, oxidative stress and inflammatory signals	Barrier disruption is associated with impaired gut function, inflammation and reduced performance	(Chelakkot et al., 2018)
Gut-associated lymphoid tissue (GALT)	Peyer’s patches, isolated lymphoid follicles, mesenteric lymph nodes, lamina propria immune cells	Antigen sampling and coordination of mucosal immune responses	Integrates immune surveillance, tolerance and adaptive responses	Microbiota development, dietary antigens, micronutrients and bioactive compounds influence maturation and activity	Supports pathogen resistance while limiting unnecessary intestinal inflammation	(Kurashima and Kiyono, 2017)
Innate immune sensing	TLRs, NOD-like receptors, C-type lectin receptors, epithelial PRRs	Rapid detection of microbial and tissue-derived signals	Activates NF-κB, MAPK, interferon and inflammasome-associated pathways	Microbial load, microbial-associated molecular patterns, dietary composition and metabolites modify signaling intensity	Appropriate activation supports defense; excessive activation can impair barrier function and productivity	(Iyer et al., 2023)
Dendritic cells and macrophages	Intestinal DCs, mononuclear phagocytes, macrophages, antigen-presentation machinery	Luminal antigen sampling and tissue surveillance	Antigen presentation, phagocytosis, cytokine production and tolerance/effector programming	Microbial metabolites, dietary antigens and inflammatory lipids influence phenotype and function	Regulates the threshold between tolerance and inflammatory defense	(Filardy et al., 2023)
Innate lymphoid cells (ILCs)	ILC1, ILC2, ILC3; transcriptional programs and cytokines including IFN-γ, IL-5, IL-13, IL-17 and IL-22	Rapid tissue adaptation, epithelial support and mucosal defense	Coordinates barrier protection, antimicrobial responses, inflammation and repair	Nutrients such as vitamin A/retinoids and vitamin D, together with microbial metabolites, influence ILC activity	Links luminal environmental signals to rapid mucosal immune adaptation	(Zelante et al., 2013; Li et al., 2023)
Adaptive mucosal immunity	CD4^+^ T cells, Th1, Th2, Th17, Treg, B cells, plasma cells	Antigen-specific defense and immune memory	Balances effector immunity with tolerance to dietary and microbial antigens	Microbial metabolites, dietary antigens, vitamins and cellular nutrient availability influence differentiation	Maintains disease resistance while preventing excessive inflammatory energy expenditure	(Kurashima and Kiyono, 2017; Allaire et al., 2018)
Secretory IgA system	IgA+ B cells, plasma cells, polymeric Ig receptor, secretory IgA	Luminal immune exclusion and microbial containment	Neutralization, agglutination, prevention of epithelial adherence and regulation of microbial communities	Microbiota composition, dietary substrates and nutritional status influence IgA production and microbial interactions	Supports pathogen control and intestinal homeostasis without strong inflammatory activation	(Mantis et al., 2011)
Microbiota	Bacteria, archaea, fungi, bacteriophages and their functional genes	Fermentation, nutrient transformation, vitamin synthesis, cross-feeding and pathogen exclusion	Educates mucosal immunity and regulates epithelial and immune-cell functions	Strongly influenced by fiber, protein, fat, bioactives, feed additives, age and environmental conditions	Influences feed efficiency, nutrient utilization, resilience and disease susceptibility	(Chen X. et al., 2022; Upadhaya and Kim, 2022)
Microbial metabolites	SCFAs, secondary bile acids, indole derivatives, polyamines and other microbial products	Molecular communication between microbiota and host tissues	Regulates epithelial integrity, T-cell differentiation, inflammatory tone and immune metabolism	Determined by substrate availability, microbial metabolic pathways and host physiology	Provides a mechanistic link between dietary composition and whole-animal physiology	(Zelante et al., 2013; Koh et al., 2016; Wahlström et al., 2016)
Epithelial–immune communication	IL-1, IL-6, IL-10, IL-17, IL-22, TNF-α, IFNs, chemokines, growth factors and antimicrobial peptides	Coordinates epithelial turnover, repair and local adaptation	Bidirectional regulation between epithelial and immune compartments	Dietary bioactives and microbial metabolites can modify cytokine and epithelial signaling	Determines whether nutritional stimuli promote repair/homeostasis or inflammatory dysfunction	(Allaire et al., 2018; Soderholm and Pedicord, 2019)
Immunometabolic regulation	mTOR, AMPK, PI3K/Akt, HIF-1α, NF-κB, Nrf2; mitochondrial metabolism	Coordinates cellular energy use, proliferation, repair and metabolic adaptation	Determines immune-cell activation, inflammatory phenotype and resolution	Nutrient availability and microbial metabolites directly influence metabolic signaling	Links immune activation with nutrient partitioning, growth, resilience and production efficiency	(Fritsch and Weichhart, 2020; Loor and Elolimy, 2022)

AMPK, AMP-activated protein kinase; CCK, cholecystokinin; DC, dendritic cell; GLP-1, glucagon-like peptide-1; GALT, gut-associated lymphoid tissue; GPx, glutathione peroxidase; HIF-1α, hypoxia-inducible factor 1α; IFN, interferon; ILC, innate lymphoid cell; MAPK, mitogen-activated protein kinase; MUC2, mucin 2; NF-κb, nuclear factor-kappa B; NLR, NOD-like receptor; Nrf2, nuclear factor erythroid 2-related factor 2; PRR, pattern-recognition receptor; PYY, peptide YY; ROS/RNS, reactive oxygen/nitrogen species; SCFA, short-chain fatty acid; sIgA, secretory immunoglobulin A; TLR, Toll-like receptor; Treg, regulatory T cell; ZO, zonula occludens.

## 3 Diet–microbiota–immune axis: molecular integration of nutritional signals and gut immunophysiology

The GIT of food-producing animals is a nutritional and immunophysiological ecosystem in which dietary inputs and microflora and host regulation interact. This relationship serves as the overarching context that nutrition effects gastrointestinal function, physiological adaptation, resiliency, and productive performance (Kogut and Arsenault, 2016; Kaore et al., 2025). The intestinal epithelium constantly receives information from the lumen of the intestine about the nutrients and microbes in the gut, while simultaneously receiving signals from the host, which is important for maintaining gastrointestinal homeostasis (Kurashima and Kiyono, 2017; Soderholm and Pedicord, 2019; Heppert et al., 2021). Therefore, the physiological effects of dietary interventions will vary based on the microbial composition, the production context, animal characteristics, developmental stage, and metabolic context, not just on the diet. The physiological effects of dietary interventions will thus depend on microbial composition as well as on the production context, animal characteristics, developmental stage and metabolic context, not just on the diet itself (Loor and Elolimy, 2022; Sharkey and Mawe, 2023). Microbial metabolism plays a key role in the availability of nutrients to the host, and microbial communities are key players in nutrient transformation and recovery (Kaore et al., 2025). Nutrient manipulation has also been shown to affect microbial communities and host immune responses in pigs with probiotic interventions (Shin et al., 2019). Another interface between nutrition and host physiology is the influence of dietary phytochemicals on host inflammatory and immunoregulatory processes (Shah et al., 2021; Shah et al., 2025). These interactions help to consider microbiota as an active functional part of gastrointestinal physiology, not as a microbial population present in the intestinal lumen. Importantly, the examples of metabolism should be considered within an experimental context. Recent reports have shown that the microbial product phytosphingosine, synthesized by *Flavonifractor plautii*, can affect glucose–lipid metabolism in humans via hepatic PPARα-associated signaling and in experimental mouse models (Li et al., 2025). This discovery is an example of microbial metabolite-host metabolic communication but is not proof of better livestock performance, feed efficiency, or animal-product quality, and should not be inferred to such outcomes without production-animal testing. Likewise, in disease models, specific polysaccharides in the diet can have metabolic effects. Blood glucose and insulin resistance were decreased, and metabolic parameters were improved in a type 2 diabetes mouse model with a water-soluble polysaccharide from *Astragalus membranaceus* (Chen X. et al., 2022). The findings do not, however, constitute direct evidence of intestinal barrier improvement or production benefits in food-producing animals as they are based on metabolic disease-model evidence. This evidence hierarchy will be invaluable for converting interactions between diet and microbiota into livestock nutrition, and prevent unsupported extrapolation from human or experimental disease models to livestock production outcomes.

### 3.1 Dietary regulation of microbial ecology and function

The dynamics of microbial populations of the gut respond dynamically to the materials that are undigested by the host and reach to the microbial niche. Typically, fermentable carbohydrates cause saccharolytic metabolism and produce short-chain fatty acids (SCFAs), while very high loading of indigestible protein reaching the distal gut induces more proteolytic fermentation, producing higher levels of ammonia, phenols, indoles, branched-chain fatty acids, etc. Microbial ecologies can be modified by dietary lipids through changes to the availability of substrate, bile secretion and the modification and metabolism of bile acids, while plant phytochemicals can exhibit selective inhibition, enrichment and metabolic transformation of a limited group of bacteria. Thus, the diet of individuals in an ecosystem creates the selection of an ecological environment where microbes interact, compete, cooperate, and evolve (Yao et al., 2016). A significant conceptual step forward in nutritional microbiology is to think that what microbes do may provide us with more information about them than what the organism is taxonomically. Different groups of microbes may have similar metabolic capabilities, resulting in functional redundancy in the community; but closely related species might react differently to the same substrates that they consume. It follows that an apparent decrease in the quantity of a bacterial taxon can’t reflect a “loss” of the beneficial function, any more than an enhancement of “specific” bacteria can correspond to a “good” effect on gut well-being. When approaching a functional assessment, the relevance of metabolic pathway analysis, substrate use, metabolite production, stability of the community and interaction with the host needs to be taken into consideration. Cross-feeding mechanisms in the microbial community mediate this functional organization. Primary fermenters can transform complex food substrates into an intermediate compound, which is taken up by a secondary community of microbes and produces metabolites with greatly altered effects as opposed to those of the dietary substrate. This enhancement, referred to as trophic interaction, contributes to the diversity of metabolites and could stabilise the metabolite network (Culp and Goodman, 2023). Dietary intervention that can facilitate appropriate substrate availability could, hence, shape the microbiota not through production of a “good” microbe, but rather through facilitating cross-feeding interaction networks in an intestinal context. These interactions are especially important in shifting situations and in the face of external adversity. The shifts in microbial community composition and metabolic substrates can result from the effects of weaning, dietary changes, high production rates, pathogen challenge or thermal stress. Hence, a robust microbiota should rather be seen as one that protects the functional metabolic output and re-equilibrates in function upon a disturbance, regardless of taxonomic continuity. Therefore, dietary interventions that sustain microbial functional stability would likely enhance gut resilience and allow de-emphasis on widespread suppression of microbes (Upadhaya and Kim, 2022).

### 3.2 Microbial metabolites as immunometabolic signals

The physiological outcomes of modulation are largely carried out through molecules produced, altered, or metabolised by gut microbes. SCFAs, secondary bile acids, tryptophan-derived indoles, polyamines, amino-acid derivatives of bacterial origin and a variety of additional species define the complex chemical space of the microbial signaling repertoire linking microbial activity and host outcome. These molecules may serve as energy substrates, receptor ligands, epigenetic modifiers or metabolic mediators linking luminal microbial metabolism and host epithelial physiology, mucosal immunity, and systemic metabolism. The SCFAs acetate, propionate and butyrate, among others, represent the main microbial carbohydrate fermentation products. In terms of biological effects, the activities extend beyond merely enabling host energy metabolism; SCFAs can activate G-protein coupled receptors (FFAR2/GPR43 and FFAR3/GPR41) and can influence intracellular transcriptomic programs, including those relating to histone deacetylase inhibition (Xu et al., 2026). Importantly, butyrate can provide an energy substrate to intestinal epithelial cells while also acting to regulate differentiation and immune-cell development and inflammatory signaling. Hence, SCFA signaling can impact epithelial metabolic fitness, antigen-presenting cell activity, as well as macrophages, T cells, and dendritic cells, among others. Nevertheless, the magnitude and Physiological effects on SCFA production are species-dependent, diet-dependent, substrate-dependent, the metabolic composition of the microbes in the intestine where they are absorbed, and the host species. The specific Architecture of the host’s intestine is one of the crucial factors to assess the effects on the microbe’s metabolomic profile. Hindgut fermentation is an important source of SCFAs, which modulate gut functions in animals with a single-compartment stomach, whereas large foregut fermentation takes place in ruminants and converts the dietary nutrients before they arrive in the small intestine. For this reason, the same dietary intervention may generate different microbial substrates, metabolite profiles, and downstream host responses across livestock species; in ruminants, foregut fermentation modifies dietary substrates and microbial metabolites before they reach the small intestine, whereas in pigs and poultry microbial fermentation occurs predominantly after gastric digestion. This adds more weight to the fact that dietary microbiome studies need to be interpreted in the context of the species being studied, their gastrointestinal system and also their production characteristics (den Besten et al., 2013). Microbial transformation of the bile acid is also another important signaling pathway. Primary bile acids are the host-produced molecules that is deconjugated and modified by microbes to create unique secondary bile acid metabolites, varying from primary bile acids. These various bile acid metabolites interact with host receptors such as the farnesoid X receptor (FXR) and Takeda G-protein-coupled bile acid receptor 1 (TGR5), resulting in a microbial influence over bile acid homeostasis, lipid metabolism, host responses in the epithelium and control of inflammatory events. It therefore follows that altering the lipid supply through diet or altering bile acid secretion or biotransformation by the microbiota may have significant effects on gut immunophysiology outside the standard digestion role of bile acids (Wahlström et al., 2016). A notable example of diet-microbial-immune communication is in dietary tryptophan metabolism. Dietary tryptophan may be metabolised by gut microbes to generate indole derivatives that activate the aryl hydrocarbon receptor (AhR), a ligand-inducible transcriptional regulator found on both epithelial and immune cells. AhR signaling plays an important role in mucosa-mediated immunity through induction of epithelial and immunological programs and has attracted much attention to the participation of group 3 innate lymphoid cells (ILC3s), a source of interleukin-22 (IL-22). IL-22 favours antimicrobial immunity, epithelial barrier integrity and tissue restitution. The Trp-microbiota-AhR-IL-22 axis therefore provides an example of how an amino acid from food can be converted by microbes in the intestine into a bioactive signal competent enough to regulate mucosal immunity. Besides affecting physiological signaling, the metabolites of microbes can even impact host metabolic signaling pathways, like the activation of the AMP-activated protein kinase (AMPK), mechanistic target of rapamycin (mTOR), hypoxia-inducible factor-1 (HIF-1α), nuclear factor-kappa B (NF-κB) and nuclear factor erythroid 2-related factor 2 (Nrf2) signaling. Through these signal ways, microbial metabolism plays important roles in altering the cellular energy status, inducing inflammatory responses, promoting oxidative defense and cellular proliferation, and promoting tissue adaptation (Zelante et al., 2013). This implies that dietary manipulation may have immunological effects indirectly through microbes’ metabolism rather than from the interaction of the diet item and immune cells. **Table 2** lists the most important dietary microbial metabolism routes and corresponding host signal pathways.

**Table 2:** Major diet–microbiota–immune signalling pathways, physiological implications, and supporting experimental models in food-producing animals.

Dietary intervention/component	Microbial or metabolic target	Host sensing/pathway	Principal immunological effect	Physiological implication	Species/experimental model supporting the evidence	References
Fermentable dietary fibre	Microbial fermentation; short-chain fatty acid (SCFA) production	FFAR2/FFAR3; histone deacetylases (HDACs)	Modulation of inflammatory and regulatory signalling	Potential support for epithelial homeostasis and fermentation efficiency	General mechanistic literature; the cited sources do not establish a single livestock species for all listed mechanisms	(den Besten et al., 2013; Koh et al., 2016)
Resistant starch/oligosaccharides	Saccharolytic microbial fermentation; SCFA production	SCFA-associated signalling	Potential modulation of regulatory immune responses	Support for intestinal homeostasis, depending on substrate and host context	General mechanistic evidence; a specific livestock model is not established by the current citation	(Koh et al., 2016)
Dietary tryptophan	Microbial production of indole derivatives	Aryl hydrocarbon receptor (AhR)–ILC3–IL-22 axis	Regulation of antimicrobial defence and epithelial responses	Potential support for barrier maintenance and tissue repair	Experimental mechanistic evidence, including mouse models; livestock-specific validation of the complete pathway remains limited	(Zelante et al., 2013; Li et al., 2023)
Dietary lipids and bile-acid metabolism	Microbial bile-acid transformation	Farnesoid X receptor (FXR); Takeda G-protein-coupled receptor 5 (TGR5)	Modulation of host metabolic and inflammatory signalling	Regulation of bile-acid homeostasis and intestinal metabolic function	Primarily general mammalian and experimental-model evidence in the cited sources; equivalent responses should not be assumed across livestock species	(den Besten et al., 2013; Wahlström et al., 2016)
Glutamate/related amino acids	Host and microbial amino-acid metabolism	Th17/Treg-associated immune signalling; STAT pathways	Modulation of Th17/Treg balance under inflammatory challenge	Potential attenuation of challenge-associated intestinal immune dysregulation	Piglets subjected to lipopolysaccharide (LPS) challenge	(Liu et al., 2023a)
α-Ketoglutarate	Immunometabolic regulation	Th17/Treg-associated signalling	Modulation of Th17/Treg balance under inflammatory challenge	Potential support for mucosal immune homeostasis	Piglets subjected to LPS challenge	(Liu et al., 2023b)
Probiotics	Microbial community and host–microbe interactions	Microbial competition and host immune/barrier responses	Modulation of intestinal barrier function, antioxidant capacity and immune responses	Potential improvement in intestinal function and resistance to challenge	Broiler chickens receiving Lactobacillus plantarum 16 and Paenibacillus polymyxa 10	(Wu et al., 2019)
Prebiotics	Selective microbial fermentation	Metabolite-mediated host signalling	Potential modulation of regulatory immune responses	Effects depend on substrate, microbial ecology and host context	The current citation supports general microbial-metabolite mechanisms but does not establish a specific livestock model for this complete pathway	(Koh et al., 2016)
Phytogenic bioactives	Microbial ecology and intestinal cytoprotective responses	Antioxidant and cytoprotective signalling; avoid assuming direct pathway activation without pathway-specific measurements	Potential modulation of oxidative and inflammatory responses	Potential support for intestinal cytoprotection and antioxidant capacity	Broiler chickens receiving dietary phytogenic supplementation	(Mountzouris et al., 2020)
Postbiotic metabolites	Microbial-derived products	Host responses to microbial metabolites; specific receptor pathways require direct evidence	Modulation of intestinal health-associated and immune responses	Potential support for intestinal function; product-specific outcomes depend on the preparation and study design	Broiler chickens receiving Lactiplantibacillus plantarum postbiotics	(Chang et al., 2022)

AhR, aryl hydrocarbon receptor; FFAR, free fatty acid receptor; FXR, farnesoid X receptor; HDAC, histone deacetylase; ILC3, group 3 innate lymphoid cell; IL-22, interleukin-22; LPS, lipopolysaccharide; SCFA, short-chain fatty acid; STAT, signal transducer and activator of transcription; TGR5, Takeda G-protein-coupled receptor 5; Th17, T helper 17 cell; Treg, regulatory T cell. Species and experimental models refer to the evidence supporting the cited pathway, not to universal validation of every molecular step in that species. Direct intervention evidence in this table includes piglets and broilers. Some SCFA-, indole-, and bile-acid-associated mechanisms are supported by general mammalian or experimental-model literature and should not be assumed to operate identically across pigs, poultry, and ruminants. Changes in microbial composition or immune markers alone do not establish functional improvement or a causal effect on production performance or product quality.

### 3.3 Microbiota–epithelial communication

The intestinal epithelium is a primary sensor of microbial activity at the host interface, mediating transformation of microbial signals into physiologic responses. Epithelial cells express innate immune PRRs, such as TLRs and NLRs, which recognise MAMPs and other signals in the intestinal lumen. The physiological purpose of PRR signaling is to enable appropriate epithelial responses to support proper epithelial differentiation, priming in preparation for encounters with microbes, and discourse with the resident microbiota. Conversely, excessive and sustained microbial sensing can activate inflammatory signaling pathways and induce epithelial dysfunction (Shah and Gupta, 2024). Critically, Host-microbe communication does not demand their complete eviction from the epithelia. A functional and healthy intestinal system necessitates regulated spatial and molecular dialogue between microorganisms and host tissues. Microbial metabolites can signal to the epithelium in ways that support energy metabolism and differentiation, upregulate epithelial antimicrobial programs, or promote epithelial repair. On the other hand, abnormal microbial life will augment epithelial damage from metabolites or signals associated with inflammatory environments. Hence, there is a dynamic feedback loop where microbe structure, function, and metabolism affect epithelial physiology, which in turn can shape the niche that the microbiome inhabits. The positioning and metabolism of microbes are also subject to the regulation of mucous secretion, antimicrobial molecules, and epithelial turnover, the levels of readily available nutrients, and the gradients of oxygen in the luminal space. As such, modifications in microbial function may also impact epithelial responses and vice versa, so that modifications in epithelial physiology will lead to the modification of the function of microbial communities. It is especially important as a potential pathobiology factor that a microbial microbial community disruption-epithelial stress-immune activation circuit may arise. Changed microbial communities or output products may stress the epithelia and trigger inflammatory signals, which in turn affect nutrient availability, oxygenation, the composition of the mucus layer and even their microenvironments and might therefore perpetuate microbial instability and the impairment of intestinal homeostasis. Dietary interventions which can maintain metabolically active microbial communities may therefore modulate epithelial physiology as a consequence of promoting desirable microbial signaling, not through direct stimulation of epithelial cells (Cai et al., 2024). Detailed structural mechanisms governing tight-junction organization, mucus architecture, epithelial permeability, and barrier restoration are considered separately in Section 4.

### 3.4 Microbiota-driven calibration of mucosal immunity

Among many roles, perhaps the most crucial for the intestinal microbiota involves its function in determining the developmental and calibration properties of mucosal immunity. Microbial stimuli are ever-present at the innate immune receptors or APCs of mucosal-associated lymphoid tissues, informing them as to the contents and metabolic status of the luminal environment. The stimuli thus act not just to stimulate immunity, but to establish at which point immunity can be stimulated, and to favour tolerance, an ability to control defensive immune responses, or inflammation. Central to the system are dendritic cells and macrophages. Microbial factors influence the Maturation of dendritic cells and expression of antigen presentation, thereby modulating T-cell differentiation. These macrophages integrate signals from the microbiota and the tissue, and are able to acquire functional states ranging from inflammatory antimicrobial effector phenotypes to tissue-supporting and regulatory phenotypes that balance mucosal tolerance, while also retaining the ability to respond to invading pathogens (Filardy et al., 2023). The balance between regulatory and inflammatory T-cell responses is especially critical; there are microbes whose metabolites, as well as antigen-presenting-cell signals, could skew differentiation of both regulatory and effector populations of T cells, such as Th17 cells. SCFAs, for instance, play a role in creating regulatory immune environments via both receptor-dependent and epigenetic mechanisms. Conversely, microbial signals together with intestinal epithelial cell signals could drive Th17- associated immune responses if such a host response needs to be mounted against infection at mucosal sites. The physiological role is therefore regulation of usage of a given pathway depending on the context, and not switching it off. Furthermore, ILC3s further bridge the connection between sensing microbes and defending epithelia: Microbial or metabolite-induced stimuli have the ability to shape ILC3 function and IL-22 production, and thereby promote antimicrobial responses and facilitate epithelial healing. This model shows how microbial ecologies can affect host immunity rapidly and innately, without classic antigen-specific T cell or B cell-adapted responses. Another dominant microbiota immune-regulating factor is sIgA; instead of just acting as a pathogen-neutralising antibody, the antibody has to aid the compartmentalisation and immune exclusion of microbes from within the lumen in a microbial ecological manner. Balanced IgA responses may inhibit invasion, while allowing tolerance of mucosal communities of commensal bacteria. Diet-induced changes in the composition of these bacteria, or function, may thus affect the number, specificity, and arrangement (functional architecture) of mucosal antibody response. Moreover, microbial sensors can also interface with inflammasome pathways like NLRP3 signaling. Under specific circumstances, inflammasome activation is beneficial to help fight off pathogens and adapt tissues to their microenvironment, but when too much inflammasome is activated, it drives the secretion of inflammatory cytokines and can ultimately lead to tissue damage; this underscores a key concept in microbiota-immune signaling: physiological benefit is obtained only through adequate signaling, not maximal immune activation (Kayama et al., 2020).

### 3.5 Microbiota–host immunometabolic integration

The impact of microbial metabolism reaches far beyond systemic immune privilege, as microbes and their products also modulate the metabolism of the host cell and integrated systemic networks. Activation, proliferation, cytokine synthesis and tissue repair consume large amounts of energy for immune responses, and systemic inflammation alters the utilization and partitioning of body nutrients among tissues or cells. Thus, metabolites offer critical links between the supply of dietary substrate and the energy and biosynthetic requirements of mucosal immunity. AMPK and mTOR offer a reciprocal network sensing nutrients to regulate cell energy status in immune responses and epithelial biology. AMPK primarily functions as a cellular energy sensor that promotes energy conservation and metabolic adaptation during energetic stress, whereas mTOR integrates nutrient and growth-factor signals to regulate anabolic metabolism, cellular growth, and proliferation. HIF-1α responds to changes in cellular oxygen availability and inflammatory or metabolic signals and promotes metabolic adaptation, including increased glycolytic activity. In parallel, microbial and inflammatory stimuli can activate NF-κB-dependent transcriptional programs that regulate immune and inflammatory responses. Crosstalk among these AMPK–mTOR, HIF-1α, and NF-κB pathways enables immune cells and intestinal tissues to coordinate cellular energy status, nutrient availability, oxygen tension, metabolic reprogramming, and inflammatory responses in a context-dependent manner (Fritsch and Weichhart, 2020). Microbial metabolites may enter directly into or indirectly feed these pathways, altering the inflammatory balance, capacity for repair, or efficiencies of metabolism. This connection is very significant in the high-producing animal where nutrient partitioning between systems is extremely narrow. Chronic inflammatory conditions of the intestine will divert nutrients away from growth, lactation, reproduction and/or deposition of muscle by increasing demands for immune support and repair. On the other hand, effective microbial metabolism and well-controlled immune functions might allow efficient functioning with reduced costs in the metabolic maintenance of homeostasis. This conceptualisation represents efficient microbiota–immunometabolic coupling. This bridges intestinal microbial ecology and whole-animal productive performance and may also explain effects beyond measures of gut health often brought by dietary manipulations (Rakhshandeh et al., 2012).

### 3.6 Dietary modulation of microbial functional resilience

The maximal physiological relevance of targeting the diet-driven microbiota may reside not in creating any given microbial ecosystem, but in creating an ecosystem resilient to perturbation. The parameters of a resilient ecosystem are defined by resistance to disturbance, functional redundancy and metabolic versatility, and resilience to the forces of disequilibrium and subsequent recovery. Such features can potentially be modulated by dietary approaches through alteration of substrate heterogeneity, fermentable carbohydrate concentration, and exposure to dietary phytochemicals, availability of specific amino acids, and other factors that determine composition. That understanding offers implications for the context and interpretation of approaches involving probiotic, prebiotic, phytogenic, postbiotic, symbiotic, and related targeting of the microbiome. The successful application of these types of methods is expected to depend on the pre-existing microbial ecosystem, animal species, age, production stage, environmental factors, and type of physiological challenge. A given microbe-treatment eliciting a beneficial outcome in one production situation may be less effective or even have an adverse outcome in another (Dogra et al., 2020). Given these facts, future nutritional strategies will gradually need to transition from nonspecific manipulation of microbiome richness and diversity towards microbiome function. **Figure 3** is a summary of causal relationships among dietary substrates, microbial ecosystem function, microbial-derived metabolites, and their cognate host sensing pathways, as well as the mucous membrane’s immune protection and physiological adaptations. This integrated model highlights that diet-induced changes in the host are mediated by a cascading series of events that are interlinked, rather than direct effects on individual taxa or immune pathways. In general, the diet-microbiota-function axis conceptualises the microbiota as an adjustable mediator whereby nutritive stimuli result in host physiological outputs; importantly, this association involves not only alterations of the microbial community composition but also the ability of nutritional interventions to remodel the host (i.e., shift microbial metabolism, influence host senses, fine-tune the immune response and modulate host nutrient expenditure). These interactions allow for exploration of the mechanistic basis upon which nutritional intervention allows maintenance of, or recovery from, a barrier defect, the chief orchestrator of mucosal integrity and stable host physiology that will be examined in the following section.

**Figure 3:**
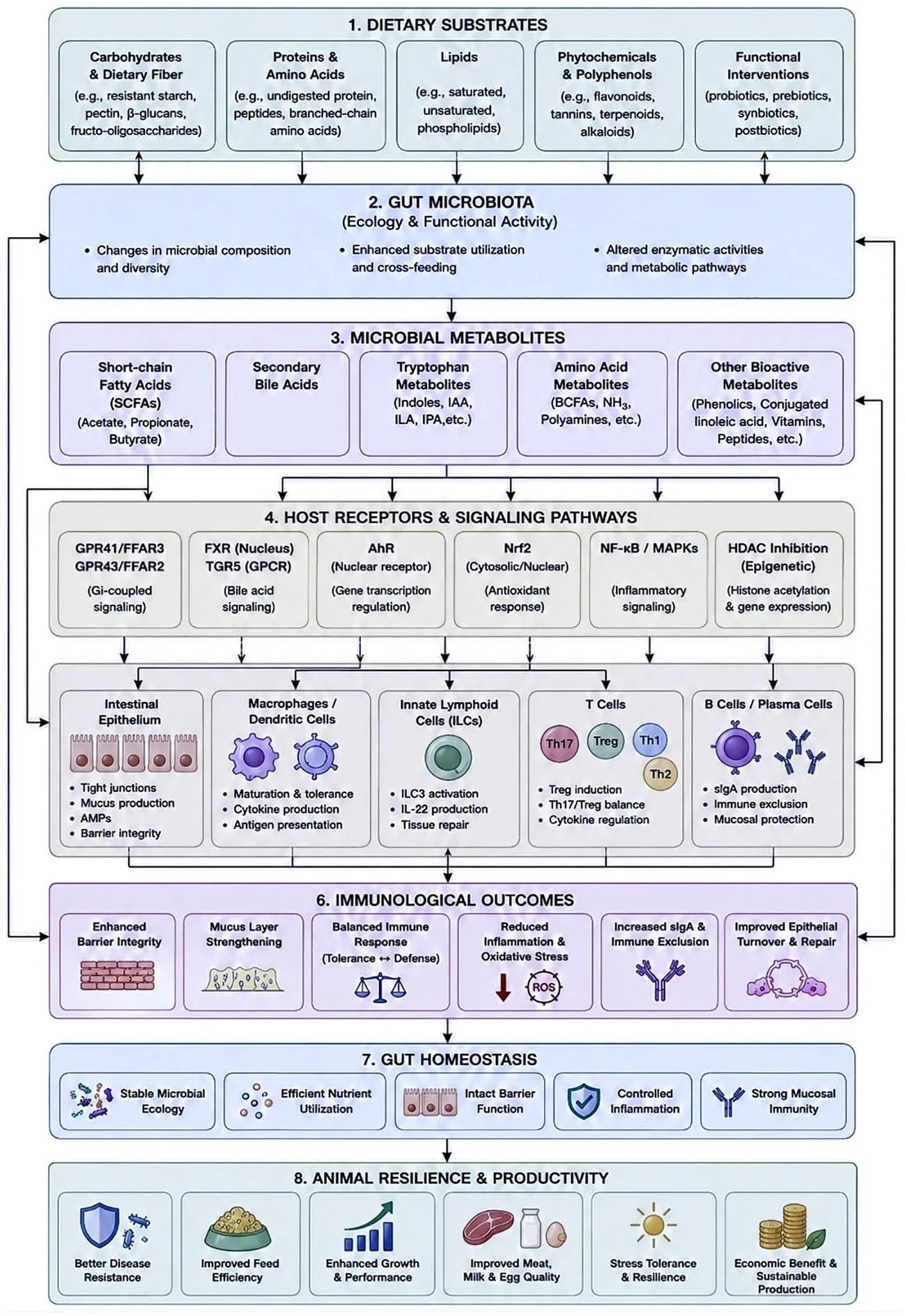
Integrated diet–microbiota–immune axis in gastrointestinal immunophysiology.

## 4 Intestinal structure and barrier defense: nutritional regulation of mucosal integrity

The intestinal barrier comprises a dynamic cellular and immune interface regulating the access of luminal nutrients, microbial factors, antigens, and pathogens to the host. This functional barrier relies on the coordinated action of the epithelial monolayer and paracellular tight and adherens junctions, the mucus layer and antimicrobial factors, epithelial regeneration, the vascular-immune system and the matrix of this epithelium. All of these factors can be affected directly by nutrient levels or indirectly by manipulation of the microbiota and its metabolic products via dietary intervention (Panwar et al., 2021). Intestinal barrier integrity may, therefore, be a useful mediator phenotype in connecting dietary modulation of gut immunophysiology to animal performance.

### 4.1 Epithelial architecture and selective permeability

The gut epithelium has to be permeable enough for the uptake of nutrients but at the same time should not allow indiscriminate access of microbes and other luminal substances. This problem is overcome by the phenomenon of selective permeability, in which the transcellular and the paracellular pathways are selective not only based on the molecular size and charge but also based on active regulation by cell signaling and nutritional necessity. The chief molecular controls of paracellular permeability are tight-junction complexes, which are comprised of claudins, occludin, zonula occludens proteins and junctional adhesion molecules. An individual’s diet can have various impacts on the morphology of the epithelium, via the regulation of cell energy metabolism, microbial fermentation, inflammatory signals and cell turnover (Chelakkot et al., 2018). There is evidence that a mixture of fermented substrates and microbial-derived substances may facilitate epithelial energy provision. The epithelial structural integrity, on the other hand, may be negatively affected by nutrient restriction, high amounts of not digested substrates, oxidative damage and inflammatory cytokines. Crucially, barrier function should not be correlated with high amounts of a single tight junction component; proper localisation, phosphorylation, association with the actin network, as well as expression of several junctional components ensures proper permeability. Recent animal data further support the combined hypothesis: Postnatal supplementation with *Lactobacillus reuteri* to lactating piglets induced parallel expression of occludin, claudin-3 and ZO-1 alongside MUC-1 and MUC-2, altered ileal morphology, suppressed inflammatory factors and enhanced protection against challenge organisms (Luo et al., 2026). Multi-omics revealed the interconnection with microbial changes and ileal metabolic profile.

### 4.2 Mucus, antimicrobial defense, and microbial spatial organization

The second crucial constituent of the intestinal barrier is the mucus layer. Produced primarily by goblet cells, mucins form a hydrated matrix that prohibits direct contact of microorganisms with the epithelial cell surface, but allows flow of nutrients and bacterial metabolites. The mucus layer also harbours antimicrobial peptides, immunoglobulins, enzymes, and other host molecules that assist the physical entrapment of microbiota. Thus, physical protection seems to be a result of not just abundant mucin, but physical attributes of mucus production and turnover, its precise positioning within the host, and its interaction with indigenous microbiota. The inappropriate proximity of microbes to epithelium would imply maximal exposure to MAMPs and pro-inflammatory factors, whereas a well-organised mucus layer provides appropriate control between host and microbes (Johansson et al., 2013). The effects of dietary interventions on the biology of mucus can be either direct (on nutrition) or indirect (on the microbiota and its signaling). Modification of goblet-cell activity and mucin expression can be achieved by fermentable carbohydrates, amino acids, phytochemicals, probiotics and postbiotic preparations. The effects of this could be especially significant when mucus production is still developing, as well as epithelial maturation, such as in developmental or stress-sensitive phases. The recent study of *L. reuteri* in suckling piglets is an example of this integrated response, showing concurrent up-regulation of mucin expression along with tight-junction proteins, microbial composition, antioxidant status and intestinal morphology (Macpherson et al., 2009).

### 4.3 Oxidative stress and barrier disruption

One of the parameters that can affect the integrity of the epithelium is the redox balance. Physiological reactive oxygen species (ROS) are involved in cell signaling, host defense, epithelial adaptation; however, overproduction of ROS can lead to oxidation of lipids, proteins, DNA, and of junctional components. Oxidative imbalance can therefore have effects on epithelial barrier functionality and on susceptibility to inflammatory and microbial insults. The barrier is a critical point of nutrition modulation, especially in poultry, which can be disrupted during heat stress with reports of increased permeability, microbial translocation, and inflammatory activation of the barrier (Liu et al., 2022). The effects of dietary antioxidants and phytogenic compounds are not simply related to their ROS scavenging capacity; rather, they can act through endogenous cytoprotective/inflammatory regulatory mechanisms. The activation of Nrf2 mediated cytoprotective programmes could be used to boost endogenous antioxidant and detoxification mechanisms while the attenuation of excessive NF-κB signalling could lead to the decrease of inflammatory amplification (Liu et al., 2022). While the elevation of the expression or activation of any of the components associated with Nrf2 or NF-κB may be taken as indirect evidence for improved barrier function, it does not necessarily signify that it is, unless backed by additional functional studies, including intestinal permeability, microbial translocation, or other complementary barrier assessments. Thus, nutritional protection of the intestinal barrier may not be limited to a simple antioxidant effect, but may include other redox reprogramming responses that are specific to species, dietary context and nature of the environmental/inflammatory challenge and that may have a different magnitude and physiological meaning.

### 4.4 Barrier failure, microbial translocation, and inflammatory amplification

In the event of epithelial disruption, luminal microbial products have greater access to the lamina propria and to the circulation. PRRs on epithelial and immune cells can be activated by LPS, fragments of bacterial DNA, fragments of peptidoglycan, microbial metabolites and other MAMPs. Eventually, through the TLR4–MyD88/TRIF, NOD-like receptor, inflammasome, and NF-κB pathways, the inflammatory mediators such as TNF-α, IL-1β, and IL-6 can be produced. One important consequence is that a positive feedback loop is set up (Iyer et al., 2023). Barrier disruption increases exposure to microbial products, triggering innate immune activation and oxidative–inflammatory injury, which further compromises barrier integrity and perpetuates a self-amplifying cycle of intestinal damage. The mechanism is important in this respect because it contributes to understanding how intestinal permeability may become a systemic physiological problem and not just a gastrointestinal abnormality. Ongoing translocation of microbial products might lead to a low-grade systemic inflammatory state, metabolic inefficiency and higher energy requirements for immune maintenance. Thus, it is important to restore barrier–immune equilibrium as the nutritional goal, instead of indiscriminate reduction of microbial exposure or inflammatory signaling.

### 4.5 Dietary strategies for barrier preservation

Multiple nutrition strategies can address aspects of the intestinal barrier biology such as the gut microbiota, intestinal epithelial metabolism, production of mucus, redox status and immune signaling pathways. Fermentable fibres and prebiotics may help improve the microbial fermentation and the metabolism of metabolic products as short-chain fatty acids, which in turn promote the metabolic function and intestinal homeostasis of the epithelium. Probiotics are able to influence microbial competition, microbial metabolite production, and epithelial–immune signaling, whereas postbiotics are more specifically microbial products that are able to interact with host pathways (Koh et al., 2016; Iyer et al., 2023). Another significant nutritional factor in barrier preservation is the amino acids. Glutamine, glutamate, arginine, and threonine may be involved in energy metabolism, mucus production, maintenance and repair of the epithelium. They can also have interactions among nutrient sensing, microbial metabolism, and immune regulation, suggesting the close linkage between intestinal metabolism and mucosal immunity in livestock (Iyer et al., 2023; Liu et al., 2024). Therefore, amino-acid nutrition needs to be considered in more than just terms of substrate provision but as a potential regulator of epithelial–immune homeostasis. Other bioactive feed additives such as plant-based phytochemicals can also be an added layer of protection by targeting oxidative and inflammatory pathways. Their effects may not be limited to direct scavenging of ROS but also involve modulation of the endogenous antioxidant defense and immune signaling. The immunomodulatory activity of phytoactive compounds has also been studied, and it suggests that they could be involved in the maintenance of mucosal immune homeostasis (Shah et al., 2021; Su et al., 2023). The use of botanical polysaccharides and their related bioactive feed ingredients also appears to be a promising nutritional approach in the context of the functional feed additives in food-producing animals. Their effects need not be extrapolated to other botanicals, as effects can vary based on compound properties, dose, bioavailability, species, developmental stage, basal diet, microbiome, and the physiological challenge. Importantly, the evidence for the effects of botanical compounds and polysaccharides is obtained under different experimental conditions and should be understood within the context of that particular experimental model. For livestock applications direct evidence from food producing animals will provide the most relevant supporting data, while disease-model studies can yield supporting mechanistic data. For instance, compounds and polysaccharides from Astragalus have been reported as modulating gut microbiota, barrier function and immune responses in the context of type 2 diabetes, but the data presented in the literature are obtained from disease-oriented studies and therefore cannot be directly correlated to food-producing animals. Likewise, *Dendrobium officinale* polysaccharide has been shown to alter gut microbiota, decrease the level of intestinal inflammation and oxidative stress, and augment the expression of tight-junction protein expression, such as Claudin-1, Occludin, and ZO-1 in a type 2 diabetes mouse model (Chen et al., 2025a). While these results are of value as mechanistic evidence for microbiota–barrier–immune interactions, the results cannot be assumed to be evidence of efficacy in livestock. Mechanistic context information can be gained from evidence from other disease models, but must be differentiated from direct evidence of production benefit. For instance, mouse models of dextran sodium sulfate (DSS)-induced colitis showed that the sheep milk diet altered the gut microbiota, SCFAs, mucin expression, barrier markers associated with the tight junctions, and inflammatory signaling (Ahmad et al., 2025; Ahmad et al., 2026b). These are observations in disease models *in vivo* and do not prove that changes in the microbiota of milk have beneficial effects on milk yield, milk composition, animal performance, or other production parameters in real dairy sheep (Ahmad et al., 2026a). The body’s reaction to any dietary change is therefore very situationally dependent. Observed effects at weaning, nutritional transition, environmental stress or other production issues could be different when production is not challenged. Nutritional strategies therefore should be considered based on specific physiological and production parameters and not be taken for granted to have a barrier enhancing effect. Dietary isobutyric acid supplementation for weaned piglets was shown to be of particular interest, as it induced beneficial effects on the intestinal mucosal barrier characteristics and at the same time modulated the cecal microbiota and serum metabolites, and improved meat-quality traits (Su et al., 2023; Buonaiuto et al., 2025; Chen et al., 2025a). The integrated response represents an important principle of nutritional gut physiology: “the modulation of intestinal barrier integrity by diet can impact on distant metabolic responses and production phenotypes.”

### 4.6 Barrier integrity as a functional biomarker of animal resilience

Intestinal morphology and barrier-associated biomarkers are increasingly being translated into measurable indicators of nutritional resilience. Intestinal health can be characterized by a combination of villus height, crypt depth, villus/crypt ratio, tight junction protein expression, mucin expression, permeability markers, inflammatory cytokines, oxidative-status indices and microbial metabolites. No one marker, however, is sufficient to effectively characterize barrier function. Increased ZO-1 expression does not necessarily mean that physiological permeability was restored, and restored villus morphology does not indicate appropriate immune regulation. Encouragingly, the ideal combination of histology, permeability, molecular markers, microbiome profiling, metabolomics and functional outcomes is preferable for integrated assessment (Perez-Diaz-Del-Campo et al., 2023). This is especially true for precision nutrition. New interventions need to be designed to identify barrier phenotypes that are predictive of weaning resistance to heat stress, infection, nutritional transition or other production challenges. These phenotypes might then provide the basis for personalised feeding plans for animals according to their age, production stage, microbiome and environmental exposure. In conclusion, the integrity of the intestinal barrier is an important point of convergence for various nutritional, microbial, metabolic and immune signals. Preserving epithelial architecture, organization of mucous secretions, selective permeability, redox equilibrium, and regeneration capacity in the diet can break the cycle of microbial disturbance to chronic inflammation and promote more efficient physiological adaptation. Diet and barrier preservation, oxidative regulation and downstream animal phenotype are summarized in **Figure 4**, and the main structural and molecular barrier targets are compared in **Table 3**.

**Figure 4:**
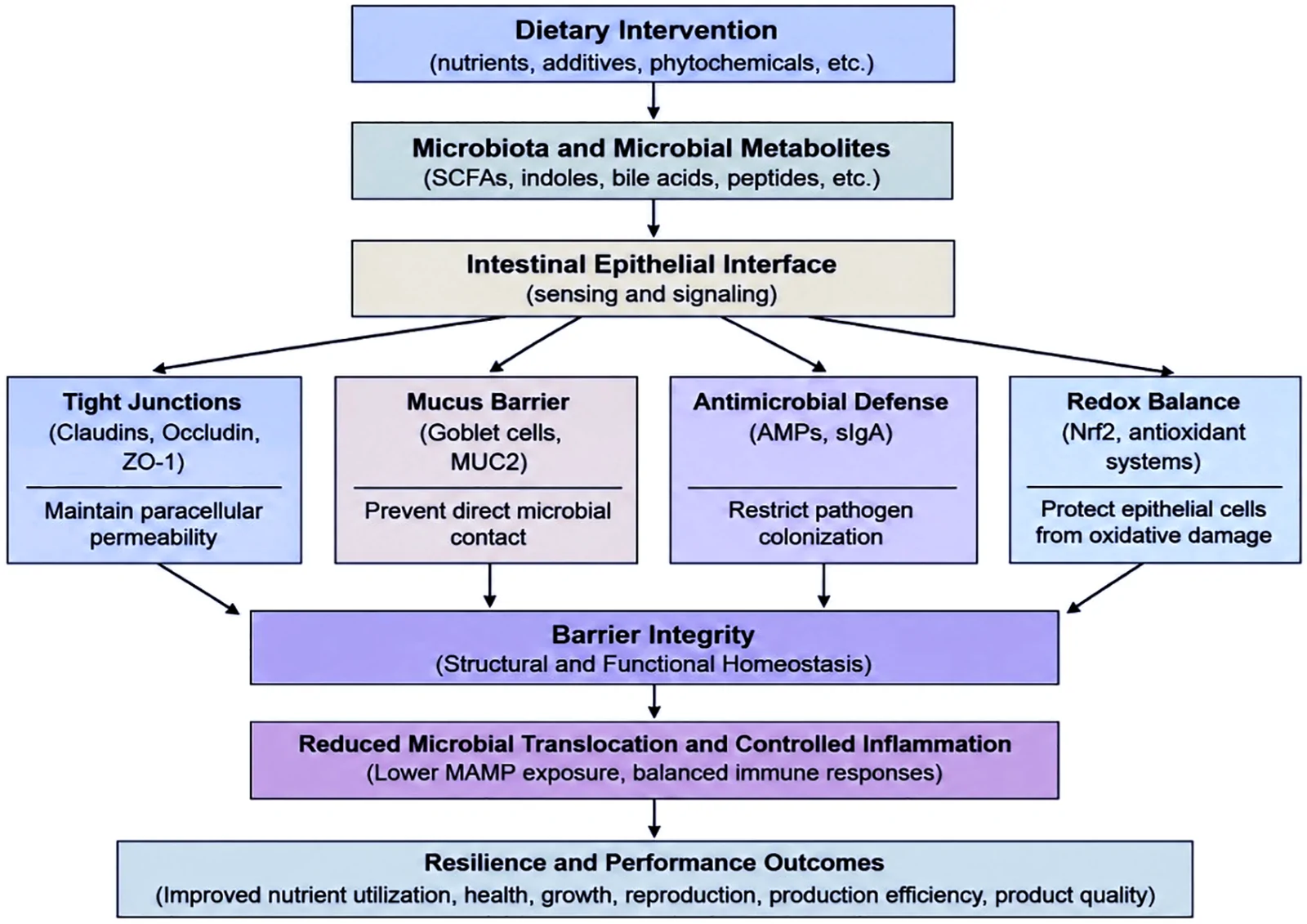
Nutritional regulation of intestinal barrier integrity and mucosal resilience.

**Table 3:** Dietary targets and mechanisms involved in preservation of intestinal barrier integrity in food-producing animals.

Dietary strategy	Primary intestinal target	Major mechanism	Expected barrier outcome	Relevant production context	Reference
Fermentable fiber/prebiotics	Microbiota	SCFA generation	Improved epithelial function	Weaning, feed transition	(Sun et al., 2021)
Probiotics	Microbiota/epithelium	Microbial competition and metabolite signaling	Tight-junction and mucus support	Early life, disease challenge	(Ma et al., 2024)
Postbiotics	Epithelial/immune pathways	Defined microbial metabolites	Barrier and immune regulation	Precision nutrition	(Zhang et al., 2024)
Glutamine/glutamate	Enterocytes/mucosa	Cellular energy and signaling	Repair and barrier maintenance	Weaning, stress	(Wu et al., 2019; Mountzouris et al., 2020)
Threonine	Mucus synthesis	Mucin production	Improved mucus barrier	High-production states	(Wang et al., 2010)
Phytochemicals/polyphenols	Redox/inflammatory pathways	Nrf2 activation, NF-κB modulation	Reduced oxidative barrier injury	Heat/pathogen stress	(Sun et al., 2021)
Organic acids	Microbiota/epithelium	pH and microbial/metabolic modulation	Improved mucosal environment	Feed efficiency, pathogen pressure	(Chen et al., 2025a)
Functional lipids	Membrane/inflammatory pathways	Lipid signaling and barrier regulation	Reduced inflammatory injury	Heat stress, production stress	(Qiu et al., 2024)

Species-context note: Representative studies in this table should be interpreted according to the animal species and physiological challenge in which the response was demonstrated. Several barrier-related interventions are supported directly by pig or poultry studies; these findings should not be extrapolated to ruminants without considering the modifying effects of foregut fermentation and species-specific gastrointestinal physiology.

## 5 Oxidative and inflammatory status in the gut: redox–immune coupling

Oxidative and inflammatory mechanisms are interrelated and regulate gastrointestinal function. Oxidative stress is not a pathological event by itself, but rather it is part of a regulatory network between oxidative stress and inflammation, where large amounts of ROS may trigger inflammatory response signaling, and activated immune cells produce more ROS and reactive nitrogen species (RNS). This redox–inflammation coupling is especially important during weaning, pathogen challenge, heat stress, and other production-related situations that increase metabolic and immunological demands. Nuclear factor erythroid 2-related factor 2 (Nrf2) is an important central molecular regulator of intestinal redox adaptation. Basal Nrf2 expression is mainly suppressed by ubiquitination and degradation mediated by Keap1. Under normal conditions, Nrf2 exists in the cytoplasm bound to a repressor and is sequestered in the cytoplasm, but oxidative or electrophilic stress releases it to allow for nuclear accumulation and binding to antioxidant response elements to induce antioxidant and cytoprotective genes. This results in induction of enzymes related to glutathione metabolism, heme degradation and cellular detoxification to help prevent oxidative injury (Wen et al., 2019). Importantly, Nrf2 signaling is intertwined with inflammatory pathways and is not just an antioxidant switch. NF-κB and inflammasome signaling are the major pathways of the reciprocal inflammatory cascade. Constant firing of pattern recognition and danger signaling pathways can lead to NF-κB-dependent transcription of inflammatory mediators and enhance cellular susceptibility to activation of the NLRP3 inflammasome. NLRP3 brings together signals from microbes, dysfunction of the mitochondria, ionic disturbances, and oxidative stress to facilitate the maturation of IL-1β and IL-18 and, under the right circumstances, pyroptotic cell death. Therefore, at the molecular level, the Nrf2–NF-κB–NLRP3 axis is a critical stepping stone in which dietary interventions can impact the shift from adaptive responses to pathological inflammation (Hennig et al., 2018). Mechanistic evidence to this concept has been provided in recent livestock studies. For weaned piglets, microbe-derived antioxidants stimulated the Nrf2–HO-1 pathway and inhibited the ROS–NLRP3–IL-1β pathway, decreasing the levels of inflammatory mediators in the intestine and enhancing growth performance (Shen et al., 2025). Dietary dihydromyricetin, similarly, enhanced antioxidant capacity and intestinal permeability in LPS-challenged piglets, while increasing the activation of Nrf2 signaling and decreasing the activation of TLR4/NF-κB, HIF-1α and NLRP3 (Chen et al., 2025b). For broilers, *Viola yedoensis* protected against LPS-induced oxidative and inflammatory damage by a coordinated regulation of both NF-κB/NLRP3 and Nrf2/MAPK pathways and changed the gut microbial structure (Zhao et al., 2024). These findings contribute to the concept of dietary antioxidants as indirect ROS scavengers, rather than direct antioxidants, and shift the paradigm to redox immunomodulation, in which the nutritional compounds boost the endogenous cytoprotection capacity while keeping the excessive inflammatory amplification in check. Of particular importance, however, are plant-derived polyphenols and other phytochemicals, as their biological action could be mediated by other mechanisms such as activation of Nrf2, protection of mitochondria, modulation of inflammatory transcription and microbiota-dependent metabolism, instead of by direct antioxidant action. The antioxidant properties of MAPK–Nrf2/ARE-associated mechanisms are also shown in recent evidence of grape pomace in broilers challenged with coccidia (Sharma et al., 2025). One of the key translational points is that oxidative signaling is a true physiological process when it is of an appropriate intensity. Full inhibition of ROS may disrupt epithelial function, such as signaling and antimicrobial defenses. Hence, dietary manipulations that restore redox balance, and not remove oxidative signaling, are the best option. The desired phenotype is one in which the gut can rapidly respond to challenge by oxidative and inflammatory mechanisms, yet be able to quickly resolve these responses. This redox–immune outlook also creates a conceptual link to product quality. Persistent intestinal inflammation and improved redox control could mediate reductions in systemic inflammatory and metabolic costs, and improved nutrient partitioning and resilience, respectively (Durand et al., 2022). Therefore, dietary modulation of intestinal oxidative and inflammatory networks could be an upstream approach to enhance animal health and production efficiency.

## 6 Gut-mediated resilience and product quality: linking immunophysiology to animal-derived foods

Finally, the practical utility of GI immunophysiology culminates in the animal’s ability to withstand and translate its response to the intestine into an efficient and productive phenotype at the whole-animal level. In food production species, resources (nutrients and energy) are perpetually balanced across maintenance, immune defense, growth, reproduction, lactation, and tissue allocation. The metabolic trade-off associated with chronic intestinal inflammation is the reallocation of resources otherwise available for production toward the support of innate and acquired immune defense mechanisms, tissue renewal/repair, and stress response coping strategies. In contrast, a metabolically sound and non-inflammatory gut environment with a functioning intestinal barrier may contribute to better utilization of feed resources for animal biomass generation and premium production of food. Gut tissue is not directly causative of productive tissue; rather through a complicated process involving the impact on available nutrients, endocrine signals, gut metabolites and local tissue metabolism. In muscle tissue, nutrient supply can contribute significantly to the deposition of protein, intra-muscle fatty acid oxidation, antioxidant capacity, and thereby the quality of meat (Fang et al., 2024). Although the growing evidence in support of a gut–muscle axis, it is important to consider the evidence available as mechanistic and associative evidence rather than as the direct causal pathway between specific taxa of microbes and meat quality. A recent review suggested that metabolites from microbes and mediators from the host, such as short-chain fatty acids, amino acids, bile acids, immune cells and hormones and extracellular vesicles, could act as mediators of gut–muscle communication and affect muscle metabolism, immune status, fibre characteristics, fat deposition and meat quality. The review also states that the mechanistic connections and the translatability of these relationships are still poorly understood. In line with this constraint, it was observed that gut microbial communities, faecal metabolites and the muscle metabolome co-correlated after fermented-feed intervention in aged laying hens (Wen et al., 2024). These correlations will be used to explore gut-derived metabolites as potential links between dietary exposure and phenotypes of the tissues, but not as evidence that changes in the gut promote improvements in muscle or meat quality. Likewise a relationship between gut microbiota and growth performance and meat-quality traits was suggested for the supplementation of broilers with dietary *Brevibacillus laterosporus*, with changes in the gut microbiota also occurring concurrently with changes in product-quality traits (Chen B. et al., 2022); however, the changes in gut microbiota did not prove to be a cause of the changes in product-quality traits. Intervention studies will be necessary to establish causality, involving targeted manipulation of specific microbial taxa or metabolites while measuring host metabolic pathways and measuring endpoints of product quality.

### 6.1 From gut health to food quality

The downstream impact or consequences of a dietary modification to the gut can be categorised into four related areas within the product-quality dimension. These areas can be considered: Nutritional characteristics, physico-chemical stability, sensorial aspects and food safety. Modifications to lipid metabolism could result in altered fatty-acid compositions. Increased antioxidant capacity of food could lead to enhanced oxidative stability. Alterations in amino-acid and microbial metabolism may influence flavour precursors, while improved gut resilience may reduce disease burden and the need for therapeutic intervention.

However, this causal link should be observed in context. Correlation with microbes and improvement in an experimental setting cannot prove a causal link and may be different from the situation that occurs during commercial food production (Koyun et al., 2022). The most compelling evidence will in large part rely on the implementation of integrated studies which link the dietary manipulation itself to the characterization of the gastrointestinal microbiome, metabolome, host immunophenotype, and in turn reflect that on tissue gene/protein expression and determine product quality (e.g. Meat, milk or eggs). This nascent, or “gut-to-food” physiology model, assumes: a direct interaction exists, such that feeding alters intra-gastrointestinal microbial activities and immune function; gastrointestinal biological changes then influence systemic nutrient allocation and tissue nutrient utilization; and ultimately, there exists a reflected phenotype measurable as an intrinsic characteristic of derived meat, milk or egg. Gastrointestinal immunophysiology becomes, in the most profound sense, central to animal resilience as well as the sustainable production of palatable and valuable animal food. In lactating mammals, gastrointestinal function affects the availability and utilization of energy, amino acids, fatty acids, vitamins and microbial compounds from dietary inputs needed in the synthesis of milk. Therefore, adjustment of gut function via dietary modification would also allow regulation of both milk yield and composition through alterations in available nutrients and metabolic status. In the same way, in laying hens, utilization of nutrients by enterocytes and utilization of microbial compounds by the bird have an impact on yolk lipid composition, mineral availability and egg quality (Doreau et al., 2020). A study in Haidong chickens reported that dietary supplementation with distillers’ dried grains with solubles altered gut microbial composition and systemic biochemical and immune indices (Bao et al., 2025). Although these findings suggest a potential link between dietary intervention, gut microbial changes, and product-related outcomes, they do not by themselves establish microbiota-mediated causality.

An increasing number of recent research results indicated that botanical feed supplements can improve many aspects of animal products, including food texture and nutritional value, not only production attributes. In late-laying hens, a multi-component botanical extract altered gut microbial profiles and was associated with increased yolk DHA, changes in the n-6/n-3 ratio, improved meat characteristics, and modification of flavour-related metabolites (Wei et al., 2025). These observations support the possibility that intestinal and ruminal microbial–metabolic processes contribute to variation in food composition and sensory phenotypes, while the causal pathways linking specific microbial changes to product quality remain to be established.

### 6.2 Integrated dietary interventions and gut-to-product nutrient partitioning

Dietary interventions that involve the GIT must be understood within a causal framework that ranges from the diet administered to intestinal function and ultimately to tissue metabolism and the phenotype of the products. This is especially relevant since the characteristics of animal products can change after dietary supplementation, whether by direct action of absorbed nutrients and bioactive compounds, by gastrointestinal function changes or by changes in microbiota-associated metabolic pathways. Probiotics, prebiotics and synbiotics are not just feed additives that “improve gut health”, but must be considered based on the biological pathways that have been shown experimentally. Probiotics can affect microbial competition, metabolite production, epithelial signaling and immune responses, while prebiotics can have effects on microbial metabolism and metabolite availability. Synbiotics combine a probiotic microorganism with a selectively utilized substrate intended to confer a health benefit. However, their effects should not automatically be assumed to be additive or synergistic, as outcomes may depend on the microbial strain, substrate specificity, dose, host species, age, basal diet, and environmental challenge (Du et al., 2023).

The first level of evidence is where effects on nutrient availability and/or tissue metabolism are demonstrated directly from diet without a microbiota-mediated pathway. The dietary supply, absorption, partitioning of metabolic pathways, deposition in tissue, and composition of products can be directly altered by changes in dietary fatty acids, amino acids, minerals, antioxidants, or other bioactive compounds. These effects can affect muscle lipid deposition or oxidative stability, the milk nutrient composition, or yolk lipid or fatty-acid characteristics without any evidence of alteration in the microbial ecology. Thus, concurrent changes in product characteristics and gut microbiota should not be interpreted as evidence that the observed product changes were mediated by the microbiota.

A second level of evidence is microbiota-associated relationships. Dietary interventions can influence the structure of the microbial community or the profile of microbial metabolites along with changes in meat, milk, or egg properties. While these observations are biologically informative, as they suggest a gut-to-tissue relationship, microbiota composition does not necessarily imply function or causality. An increase in the abundance of a microbial taxon or a change in diversity or changes in fecal microbial profiles alone do not prove the production of the downstream product phenotype by microbial metabolism. This is especially relevant when using fecal samples for studies, as the fecal communities may not reflect the entire microbial communities and/or metabolic processes at certain gastrointestinal locations. A third and higher level of evidence comes when a microbiota-mediated pathway is demonstrated through experimentation by combining microbial, metabolic, host and product measures. For example, if a dietary manipulation is shown to result in a change in microbial function or a change in the production of microbial metabolites, then a corresponding change in intestinal signaling, systemic nutrient metabolism, tissue phenotype, and finally product characteristics would provide much greater evidence for a gut-mediated mechanism. In light of the emerging livestock studies that have examined the connection between microbial profiles, the production of microbial metabolites and muscle metabolic phenotypes, a gut–muscle axis seems to be a plausible model, although the causal role of intestinal microbial processes is still not fully understood. Likewise, relationships between the intestine and nutrient utilization are associated with availability and metabolism of substrates for milk production in dairy animals and yolk deposition and egg formation in laying poultry and should not automatically be considered as microbiota-mediated effects. The same evidence hierarchy applies to product quality. Changes in lipid metabolism may influence fatty-acid composition, whereas redox status may affect oxidative stability. Amino-acid metabolism and microbial-derived metabolites may also contribute to flavour-related compounds and other sensory attributes. These outcomes should be interpreted as diet-associated effects unless the specific contribution of a microbiota-mediated pathway has been experimentally demonstrated (Chen B. et al., 2022; Wen et al., 2024; Bao et al., 2025; Wei et al., 2025).

Recent livestock studies that linked dietary approaches with interventions using either the microbial or botanical components of the diet to the characteristics of meat and eggs therefore, have evidence of diet-associated product modulation, but not necessarily evidence that the observed product phenotype was mediated through the gut microbiota. Longitudinal studies that combine microbial exposure, microbiome composition and function, metabolomics of microbes, intestinal permeability and immune phenotyping, systemic nutrient partitioning, tissue metabolism, and direct assessment of meat, milk, or egg quality would be useful to improve causal inference. If available, interventions that specifically target microbiota manipulation or mechanistic perturbation would further differentiate correlation from causation. Thus, the gut-to-product concept should be considered as a testable physiological pathway rather than a universal pathway. There is evidence to suggest that the intestine is a key organ of substrate regulation of the microbiome and that a link exists between diet and the gut’s epithelial and immune function, nutrient partitioning and downstream tissue phenotypes. The contribution of direct dietary effects, host-mediated responses and microbiota-dependent mechanisms are, however, still species-, stage- and environment-dependent. This distinction is particularly important when providing a link between intestinal immunophysiology and animal resilience and the nutritional, physicochemical and sensory attributes of animal foods (Gustafsson and Johansson, 2022). What is clear from this review is that diet can impact the intestinal and systemic phenotypes at multiple partly overlapping layers of biological regulation. While changes in the microbiota may be sufficient to explain downstream changes in production traits, the evidence can be grouped by molecular targets that include epithelial barrier proteins, inflammatory signaling, antioxidant and cellular-protective responses, and immunoregulatory pathways. In **Figure 5**, these molecular domains with the functional responses of the intestine and outcomes at the host level are combined. Notably, the molecular profiles displayed are not experimental measures, but a conceptual evidence synthesis, meant to illustrate the molecular responses described in the literature and not to be understood as a single experimental dataset or as uniformly demonstrated in livestock species.

**Figure 5:**
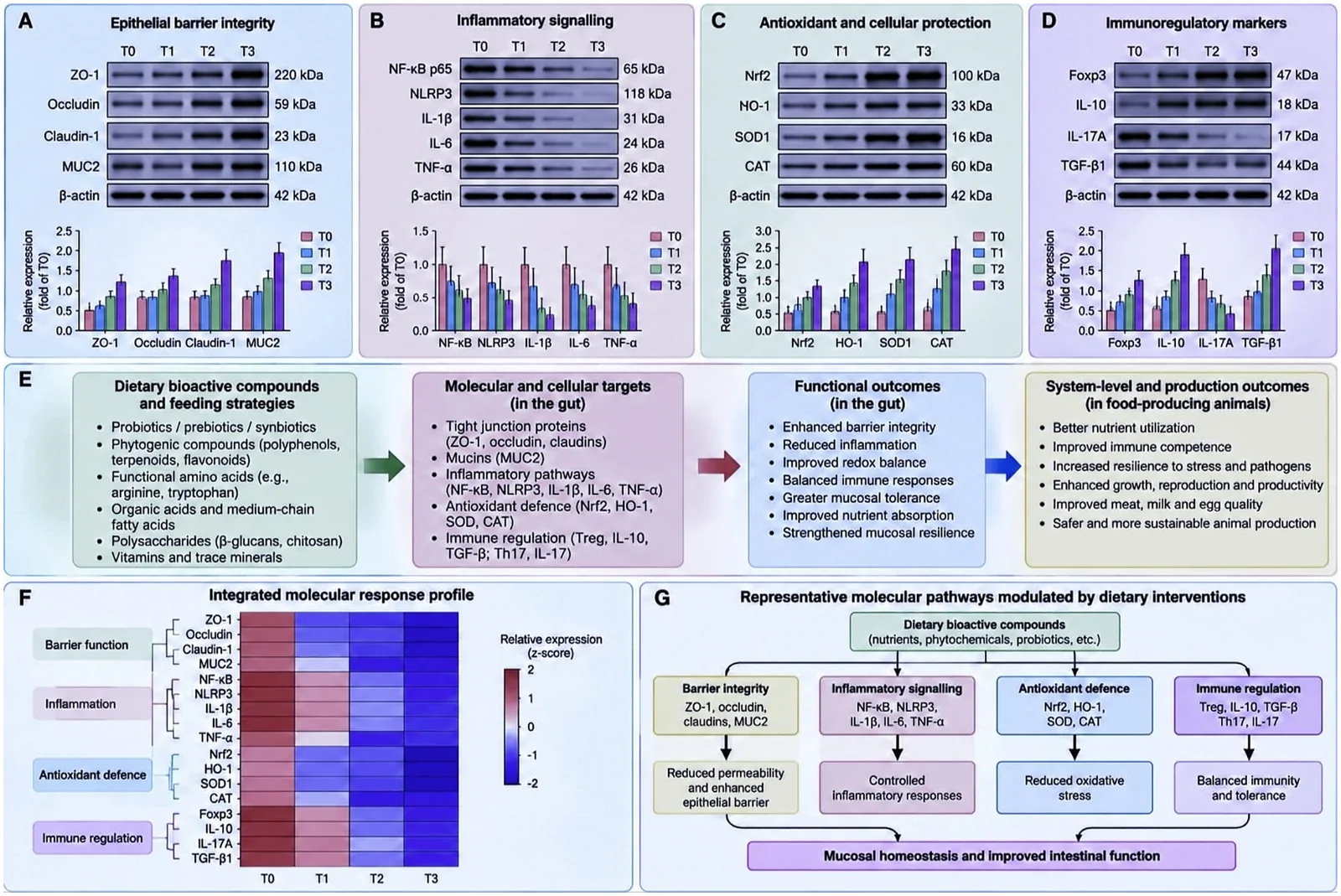
Integrated molecular evidence landscape linking dietary interventions with gut immunophysiology and downstream physiological outcomes. **(A–D)** Conceptual blot-style representation of epithelial barrier, inflammatory, antioxidant, and immunoregulatory molecular responses; **(E)** dietary intervention–molecular target–intestinal function–host outcome continuum; **(F)** integrated molecular-response heatmap; and **(G)** representative pathways linking dietary signals to mucosal homeostasis. The figure synthesizes reported molecular responses across the literature and is conceptual rather than an original experimental dataset; pathway strength and biological relevance remain species-, context-, and evidence-dependent.

### 6.3 Critical appraisal and limitations of the evidence

Experimental design, the variety of species used, the choice of outcomes and the level of mechanistic resolution of the evidence linking dietary interventions to gut immunophysiology vary. Changes in microbial composition, intestinal biomarkers or immune parameters are often observed in response to dietary interventions, but they do not always indicate functional improvement or cause the whole animal to perform better. Data on direct intervention in pigs and poultry are more advanced than in ruminants, while comparable mechanistic data in ruminants are less well represented and have to take into account foregut fermentation and species-specific nutrient processing. The interpretation is further complicated by differences in the age of the animals, the stage of production, basal diet, dose and duration of the intervention, and environmental challenge and experimental conditions. The location of sampling, as well as the methods used for sequencing, analysis and definitions of intestinal health also differ when studying the microbiome. It is therefore important to note that relative taxonomic abundance, predicted metabolic capacity and measured microbial activity should not be considered as the same type of evidence. In a similar way, fecal microbial characteristics might not reflect the characteristics of the associated mucosa and/or microbial processes within individual compartments of the gastrointestinal tract, and metabolite levels alone do not indicate the site of production or biological significance. Another restriction relates to causal inference. Simultaneous alterations in microbial community structure, immune responses in the gut, nutrient partitioning, and product attributes are insufficient to establish the link between dietary exposure and end phenotype in microbiota-mediated processes. Absorbed nutrients and bioactive compounds can have direct effects, host-mediated effects, or microbial effects, which may occur simultaneously. Incorporating studies on the integrated dietary intervention that take into account microbial composition and function, metabolite measurement, intestinal and systemic phenotyping, tissue level metabolic assessment, and direct production or product quality outcomes will improve the level of causal evidence. Mechanistic perturbations and independent testing of proposed pathways should be done where possible. Last but not least, rodent, human and cellular system findings are useful for establishing biological plausibility, but are not necessarily interpreted as demonstrated responses in food-producing animals. Species specific validation, standard phenotyping, long term and function oriented microbiome studies and controlled studies under commercially relevant conditions should be given priority in future research. The diet–microbiota–host–product framework is thus a convenient tool to structure existing evidence, but predictive and translational potential is limited until further mechanistic and intervention-level validations are demonstrated.

## 7 Conclusion and future perspectives

The dietary modulation of gut immunophysiology is a means to comprehend the interactions between nutritional inputs and gastrointestinal homeostasis, mucosal immunity, nutrient utilization, animal performance, and quality of animal-derived foods. The gastrointestinal tract is a unified nutritional-microbial-epithelial-immune-metabolic interface in which dietary substrates can act on the microflora, on the production of metabolites, on epithelial integrity, on the immune system response of the mucous membrane, on the redox balance of the tissues and on the partitioning of nutrients in the body. Hence, nutritional management should not just aim at boosting diversity of microorganisms or stimulating immune activity (Li et al., 2023; Liu et al., 2023a). Instead, it should promote resilience of the gut, integrity of gut barrier function, proper immune tolerance, ability to respond to physiological and environmental stress and return to homeostasis. This stability can decrease costs of long-lasting inflammation and aid in nutrient distribution to growth, reproduction, lactation, and productive processes (Wu et al., 2019; Liu et al., 2023b). Such responses are species-specific, depend upon gastrointestinal physiology, production stage, basal diet, microbial ecology, health status, and environment. What you find in one livestock system cannot be translated to another. Dietary changes can affect the profile of microbiota, immune markers, nutrient partitioning and/or the characteristics of meat, milk or eggs, which can occur simultaneously, but do not provide evidence for microbiota-mediated actions. There are no consistent causal connections between gut microbial processes and the attributes of animal products. Longitudinal microbiome and metabolome studies, intestinal and systemic immunophenotyping, and tissue-level metabolic measurements with direct product quality assessment would improve causality inferences in integrated intervention studies (Mountzouris et al., 2020; Sun et al., 2021; Chang et al., 2022). Gastrointestinal sampling location and analytical techniques should also be considered in such studies, as microbial flora in the feces may not reflect the flora or function (or metabolite production) at a particular intestinal site. Microbiome profiling, multi-omics, sensor-based phenotyping, artificial intelligence and predictive modelling are potential tools for precision nutrition and should not be considered routinely validated until further validation efforts have been conducted. All decisions regarding nutrient requirements, species physiology, production stage, environmental conditions and experimental responses should continue to be based on established nutrient requirements (Ma et al., 2024). New technologies can help with the discovery, biological characterization, monitoring and decision-making support of biomarkers, although it is important to validate them proactively and independently for them to be of practical use (Wang et al., 2010; Zhang et al., 2024). Validation should evaluate predictive accuracy, calibration, intra-reproducibility and inter-reproducibility, robustness and temporal stability between breeds, diets, developmental stages, production systems and environmental conditions. The effectiveness of model-based dietary decisions for repeatable animal health and welfare benefits, feed efficiency, growth, production performance, product quality and environmental efficiency should also be demonstrated through controlled intervention trials (Paraskeuas et al., 2024; Qiu et al., 2024). The economic feasibility and performance under commercial conditions also need to be assessed. More importantly, if a diet is chosen based on the correct prediction of an animal phenotype, this is not enough proof to claim that that diet will enhance the phenotype. Going forward, research should move from descriptive microbiome profiling to mechanistically driven and experimentally proven diet–microbiota–host models. The translational route should involve discovery of biomarkers, testing of mechanisms, independent validation of predictive ability, recruitment of prospective intervention trials, and commercial evaluation. The ultimate goal is not the manipulation of the microbiome itself, but development of reproducible, species-specific nutritional strategies that promote intestinal resilience, animal health and welfare, resource efficiency and high-quality animal food production, with sustainable production practices.
